# Novel Method for Realistically Simulating the Deposition of Thin Films from the Gas Phase and its Application to Study the Growth of Thin Gold Film on Crystalline Silicon

**DOI:** 10.1021/acs.jctc.5c00319

**Published:** 2025-04-30

**Authors:** Szymon Winczewski, Jacek Dziedzic, Marcin Łapiński, Jarosław Rybicki

**Affiliations:** † Faculty of Applied Physics and Mathematics, Gdansk University of Technology, Narutowicza 11/12, Gdańsk 80-233, Poland; ‡ School of Chemistry, University of Southampton, Highfield, Southampton SO17 1BJ, U.K.; § TASK Computer Centre, Gdansk University of Technology, Narutowicza 11/12, Gdańsk 80-233, Poland

## Abstract

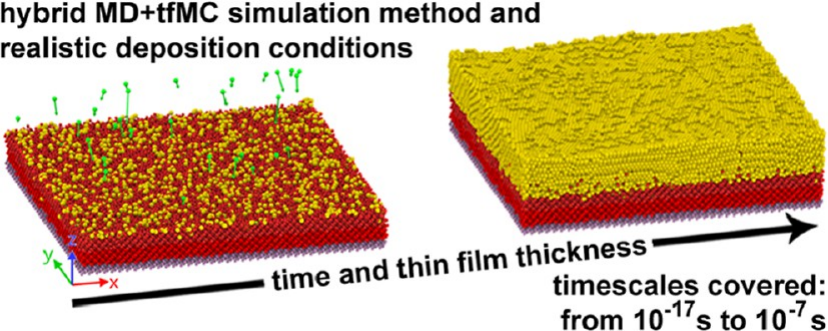

We present a novel approach for simulating thin film (TF) deposition from the gas phase at the atomistic scale, combining molecular dynamics (MD) and time-stamped force-bias Monte Carlo (tfMC). In this approach, MD, with its fine temporal resolution, captures fast events, such as incident atom-substrate collisions, while tfMC simulates slow relaxation processes, enhancing temporal scale coverage. The proposed approach also adequately models deposition conditions, for example, by accounting for realistic energy and angle distributions in the description of the incident flux. To demonstrate its efficacy, we apply it to simulate the physical vapor deposition of a 3 nm Au TF on crystalline Si. We find that the entire deposition process consisted of four distinct stages: (i) the initial degradation of the Si substrate, (ii) formation of a mixed Au–Si interface layer, (iii) nucleation and growth of a polycrystalline Au layer, proceeding in a fashion close to the Frank-van der Merwe mode (layer-by-layer growth), and (iv) postdeposition relaxation of microstructure. The produced TF was comprehensively characterized, revealing that the deposited polycrystalline Au layer contained a considerable number of defects, including dislocations, stacking faults, grain boundaries, and Si impurities. The analysis also showed that in the simulated high-energy deposition the Si substrate was considerably degraded and that the disordered Au–Si layer which formed at the interface resembled the melt-quenched Au_82_Si_18_ eutectic. A comparison with an analogous MD simulation revealed that the MD + tfMC approach extended the accessible time scale 5-fold, allowing us to reach the microsecond scale, and yielding a TF with higher crystallinity and better-developed microstructure. The deposition rate used in the MD + tfMC simulation was two to 3 orders of magnitude lower than in other recent, but purely MD, simulations, being significantly closer to experiment.

## 1 Introduction

The properties of thin films (TFs) often differ considerably from those of bulk materials of the same composition. By offering new functionalities and capabilities, TFs find applications in many areas, such as electronics, optics, electrochemistry, and sensors, attracting continually growing attention.^1,2^

It is well-known that the properties of TFs strongly depend on how they are fabricated. However, the limitations of experimental techniques (particularly, those of spectroscopic and imaging methods) hinder a better understanding of the mechanisms governing the growth of very thin films, between a few and a dozen nanometers thick. This, in turn, hinders understanding of how the manufacturing process translates into the characteristics of the TF and its resultant properties.

Physical vapor deposition (PVD) is a class of techniques used for manufacturing TFs. The distinguishing feature of all PVD techniques is that the deposited material transitions from the condensed phase to the vapor phase and then back to the condensed phase. Therefore, the PVD process involves three steps, which are (i) desorption of the material to be deposited from the source (within PVD, this is achieved utilizing physical processes, for example, by thermal evaporation or by bombarding the sputtering target with high-energy ions), (ii) transport of the atoms desorbed from the sputtering target to the substrate to be coated, and (iii) deposition of the coating on the substrate surface.

**Figure 1:**
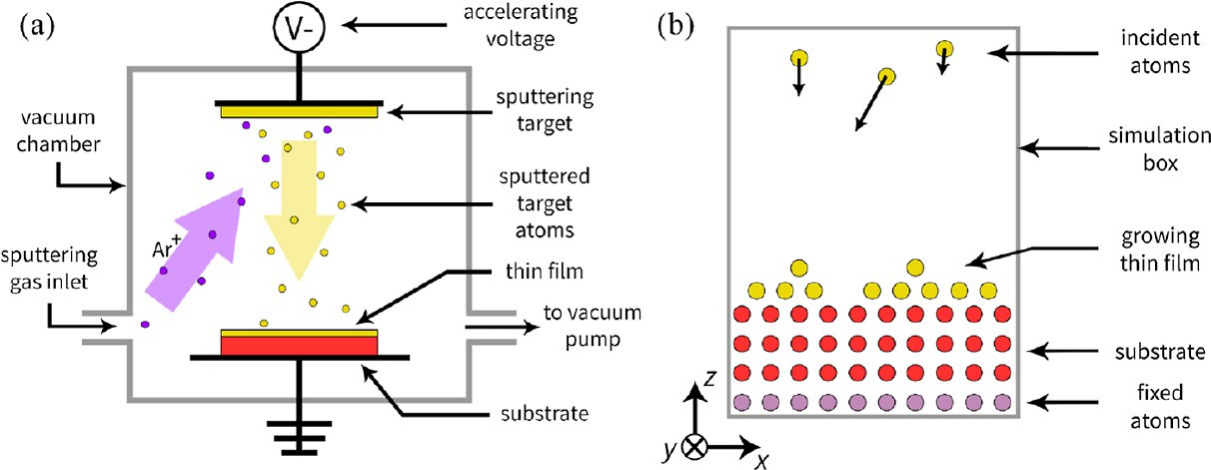
Physical vapor deposition: (a) typical experimental setup for ion beam sputtering and (b) the system used in an atomistic simulation.

Figure 1a presents a typical experimental setup for carrying out the PVD with ion beam sputtering. In a vacuum chamber, ionised particles of the sputtering gas (for example, Ar^+^) are accelerated toward the sputtering target by, e.g., an applied electric field. Their high-energy collisions with the sputtering target cause ejections of its atoms. These atoms later travel through the chamber, arrive at the substrate, and preferably deposit on its surface, causing a growth of the TF.

Atomistic modeling techniques allow studying phenomena at the atomic scale and, as such, provide tools for studying the deposition of TFs from the gas phase. Atomistic-based modeling (see Figure 1b) typically focuses on the phenomena that occur during the last step of the PVD process at the substrate’s surface, modeling the deposition-induced growth as a series of collisions of the arriving incident atoms with the substrate. In such an approach, the initial velocities are assigned to the incident atoms, chosen such that their motion is directed toward the substrate. The collisions of the incident atoms with the substrate are modeled by explicitly determining the trajectories, and for this purpose, the molecular dynamics (MD) method is typically employed (for examples, see refs (3–25)). MD can provide a detailed picture of the incident atoms’ dynamics and, in principle, of all the processes occurring in the growing TF and substrate. By repeating the above procedure—adding new incident atoms and finding their trajectories—one may observe the resultant growth of the TF in a simulation. However, such an approach suffers from a number of serious drawbacks which originate from the limitations of MD and other simplifications and approximations that need to be introduced to make the simulation computationally feasible.

In MD numerical integration is used to find the time evolution of the simulated system. This imposes a requirement of using a sufficiently small time step (typically in the order of fs), limiting the accessible time scales. These typically do not exceed several nanoseconds for system sizes under consideration here, even when supercomputing resources are used. Consequently, the growth rates used in MD deposition simulations are necessarily extremely high, exceeding those typical for experiments by several orders of magnitude. The short time scales mean that some effects, such as long-time diffusion or microstructure formation, cannot be observed in MD simulations of TF deposition. The underlying reason is that the thermodynamic driving forces for these effects are weak, which requires times that are considerably longer than those accessible to the MD simulation.

The above problem can be circumvented by combining the MD method with another simulation technique. In such a simulation protocol, the collisions of the incident atoms with the surface are simulated with the MD method, while a second technique—which should offer better phase-space sampling—is used to model all the phenomena occurring between the collisions on significantly longer time scales. The two techniques are used alternately in a cycle to model the continuous growth of the TF. Such an approach was used to study the deposition of a SiO_2_ thin film by Taguchi and Hamaguchi,^26^ who showed that combining MD with the Monte Carlo (MC) method yields more realistic results, with the TF’s morphology in better agreement with experiment. Similar simulation protocols were also used by other authors.^27,28^

It should be emphasized that the basic (i.e., Metropolis) MC method is, however, not the best choice if one is particularly interested in extending the time scale of a simulation. Better choices mainly differ in the construction of the random moves and the criteria used for their acceptance (for an overview, see e.g. ref (29)). It is worth noting that the random moves constitute the essence of any MC algorithm, with its performance depending first and foremost on the ability of the random moves to efficiently explore the configurational space.

The time-stamped force-bias Monte Carlo method^30^ (tfMC) is an example of a modified MC technique. The basic idea behind the tfMC method is to use the information concerning the forces acting on the atoms to construct random moves. Adding such a deterministic bias allows the algorithm to sense the potential energy landscape, making it more effective in overcoming energy barriers. This, in turn, improves the probing of the configurational space and helps with the exploration of rare events.

The use of forces also allows associating a time scale with the tfMC simulation. The corresponding effective time step (which is a theoretically derived statistical time corresponding to a single tfMC step) is given as^30^
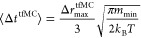
1Here, *m*_min_ represents the mass of the lightest atom present in the simulation, *k*_B_ and *T* represent the Boltzmann constant and temperature, respectively, and Δ*r*_max_^tfMC^ represents the maximum allowed displacement in the random moves taken, a parameter of the tfMC method. Although recent work^31^ postulated a different dependence of ⟨Δ*t*^tfMC^⟩ on Δ*r*_max_^tfMC^ (suggesting that ), this new estimation found that taking larger Δ*r*_max_^tfMC^ still results in longer ⟨Δ*t*^tfMC^⟩ and, therefore, longer time scales reached in the tfMC simulation. However, the Δ*r*_max_^tfMC^ parameter cannot be arbitrarily large, as for too large Δ*r*_max_^tfMC^, the detailed balance principle will be violated. Refs (30 and 31) suggested that the correct Δ*r*_max_^tfMC^ values should not exceed 5% to 10% of the shortest interatomic distance. This necessity to use small Δ*r*_max_^tfMC^ limits the simulation speedups which can be obtained with the tfMC method. As demonstrated in refs (31 and 32), these speedups are system- or process-dependent, ranging from none (diffusion in liquid state) through two to 3 orders of magnitude (solid-state processes such as crystallization) up to several orders of magnitude (healing of point defects in graphene).

From the formal point of view, the tfMC method is a uniform-acceptance technique, i.e., a method in which each step taken is accepted with unit probability. What also distinguishes the tfMC method is that instead of single-particle moves (which are very common in MMC simulations), it uses collective moves, displacing all atoms within a single step. Both of the above increase the efficiency of configurational space sampling and make the computational cost of a single tfMC step to be—in practice—comparable to that of an MD simulation. Among the advantages of the tfMC method are its formal simplicity, elegance, ease of implementation and application.

The advantages of the tfMC method were demonstrated in many recent papers which focused on various nanoscale systems and phenomena which involve slow relaxation, like the formation and processing of different carbon nanostructures (nanotubes,^33−36^ graphene,^37−40^ activated carbon,^41,42^ nanocrystalline carbon^43^), growth of Au nanoclusters,^44^ melting of Ni–C nanoclusters,^45^ and the oxidation of Si nanowires.^46^ These works demonstrated that the hybrid MD/tfMC approach—when combined with an adequate description of the interatomic interactions—can provide a reliable picture of the system’s long-time evolution, over time scales reaching microseconds.^31,33,46^

It is worth nothing that other methods can be employed to extend the accessible time scales in simulations, such as temperature accelerated dynamics,^47^ parallel replica dynamics^48^ (PRD), parallel replica tempering,^49^ bond boost,^50^ collective variable-driven hyperdynamics^51^ (CVHD), or hybrid approaches such as PRD + CVHD.^52^ However, these techniques are highly advanced and their application often requires verifying numerous assumptions and/or determining a large (and often nontrivial) set of parameters, as well as performing careful and often extensive validation. In contrast, the tfMC method is characterized by considerable simplicity, both formally and in implementation, with its configuration reduced to specifying only a single parameter, namely the maximum step length Δ*r*_max_^tfMC^. This, combined with numerous examples demonstrating the exceptional efficiency of tfMC in accelerating surface processes and nanostructure growth,^33−46^ led us to select tfMC for our simulations.

It must be pointed out that the time scale problem does not constitute the sole difficulty, and realistic modeling of deposition must also account for other important aspects. For instance, it is known that the parameters of the incident atoms (such as their energies and incident angles) follow some distributions. Although these are often known (from theory or experiment), they were often ignored in simulations carried out to date (see, e.g., refs (11,13,20,21,24,25 and 53)), even though they may influence the morphology and properties of the deposited TF.^12,14−16,19^ For example, high-energy collisions may stimulate surface diffusion, promoting surface relaxation. Accounting for the potential implantations (possible in high-energy collisions) may also change the character of the interface layer between the substrate and the deposited TF, altering its bonding.

Simultaneously accounting for the high- and low-energy incident atoms within a single MD simulation poses additional problems due to the need to perform numerical integration of equations of motion that would be at the same time accurate, and computationally efficient for systems containing both very fast and very slowly moving atoms. We identify these difficulties as one of the reasons why the deposition simulations carried out to date neglected the energy distribution.

In this work, we developed a new method for simulating the growth of TFs obtained through deposition from the gas phase. The proposed approach combines MD and tfMC to extend the time scale accessible in the simulation. It also accounts for other aspects essential for realistic modeling, as outlined above. After presenting the method (Section 2), we applied it to study the deposition of thin Au film on crystalline silicon (details are given in Section 3). This allowed us to demonstrate the practicability of the proposed approach (results are presented in Section 4) and to investigate its properties (Section 5). Among other things, we quantified the real benefits of the proposed simulation protocol, comparing it to a conventional, MD-only approach. We conclude with a summary in Section 6.

## 2 New Method for Simulating Deposition

### 2.1 Overview

**Figure 2:**
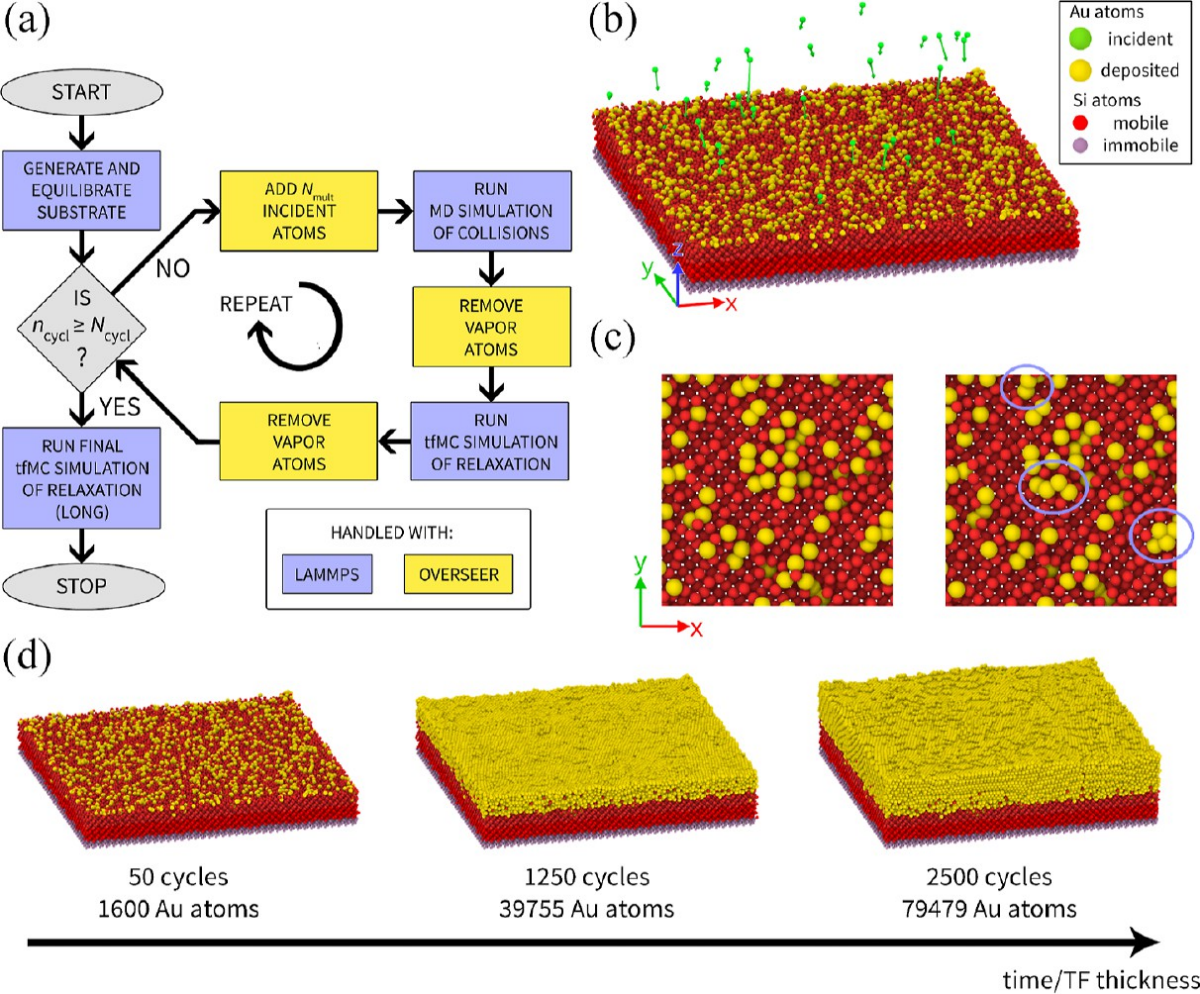
Proposed novel approach to simulating deposition. Panel (a) presents the flowchart of the simulation protocol, which combines MD and tfMC. Panel (b) shows a snapshot of the simulated system. In this case, the deposition of a 3 nm Au film on the crystalline Si was simulated (details in Section 3.1). Here we show the system’s state at the start of cycle #50. The green spheres represent *N*_mult_ = 32 incident Au atoms, which were added to the system and whose deposition was simulated in the MD run that followed. Green arrows depict their initial velocities, generated randomly according to eqs 3 and 4. Panel (c) shows a top view of a fragment of the system surface, presenting the relaxation observed in the subsequent tfMC run. The two configurations shown correspond to its beginning (left) and end (right). In the latter, circles guide the eye, denoting the Au atoms whose positions changed significantly. Panel (d) presents the picture obtained from the entire simulation, showing the system near the beginning, middle, and end of the growth cycle.

Figure 2 presents the flowchart of the proposed simulation method (a) and a visualization of the simulated system (b). Within the proposed method, a fragment of the substrate’s surface is considered with a specified orientation. Periodic boundary conditions are applied in both in-plane directions (*x* and *y*), such that the simulated system is a quasi-infinitely extended surface.

The substrate atoms from the lower part are fixed in their perfect crystalline positions. These atoms simulate the influence that the bulk of the substrate exerts on its surface, e.g. by constraining the in-plane dilatation. The remaining atoms are free to move, and their dynamics is modeled explicitly.

The growth of TF is modeled by simulating the deposition of the individual incident atoms arriving at the substrate. Their collisions are simulated using MD, which can reproduce all the related dynamical effects, such as deposition, reflection, implantation, and resputtering. MD is also used to simulate the fast relaxation occurring immediately after collisions, e.g. due to the propagation of phonons induced in the collisions.

There is another type of relaxation that occurs between infrequent collisions as a consequence of, e.g., surface diffusion. This slow relaxation is also explicitly modeled, and the tfMC method is used for this purpose. The use of tfMC considerably extends the accessible time scales, which would otherwise be very limited in a purely MD simulation.

Simulation starts with the preparation of a substrate. In this preliminary stage, the generated substrate is subjected to an MD run, carried out under constant volume and constant temperature conditions (*NVT* ensemble). The goal here is to equilibrate the substrate. The atomic positions and velocities obtained from this run are used as the initial condition in the first deposition cycle that follows.

The main part of the simulation protocol (loop over cycles, see Figure 2a) consists of a series of MD and tfMC runs. These runs form a continuous simulation because the final atomic configuration obtained from a run is used in the subsequent run as the initial structure.

At the beginning of each cycle (loop iteration), a given number of incident atoms (*N*_mult_) is generated above the substrate. Their collisions with the substrate are later simulated in the MD run. In the following tfMC run, long-time relaxation is addressed. The so-obtained atomic configuration is used in the next cycle, which adds and deposits the next portion of *N*_mult_ atoms. The entire procedure is repeated *N*_cycl_ times, until a TF of a given thickness is grown.

Because of the sequential character of the algorithm, in the absence of any further refinement, the behavior of the atoms deposited in the later cycles would be simulated over limited time, compared to the atoms deposited earlier. To mitigate this, in our protocol, the simulation does not stop after depositing the last batch of the incident atoms, and the obtained TF is simulated for some additional time in the final tfMC run. This run is intended to relax the atoms deposited in the later cycles, guaranteeing that the surface of the TF is also adequately relaxed.

The proposed protocol also contains two stages in which vapor atoms are removed. This procedure is carried out after each MD and each tfMC run and was introduced to avoid excess vapor accumulation, a problem that could otherwise arise in the adopted approach. Details of this procedure will be given later.

An approach that combines the MD and tfMC methods was already used in the literature,^33−36,38,39,45,46^ also for simulating deposition.^53^ The distinguishing feature of our method is that it better reflects the conditions of real deposition experiments. This was achieved by accounting for many physical aspects, which we believe are critical, but were to date ignored or treated without the diligence they deserve. Taking them into account required applying several problem-oriented algorithms. All of the above, i.e., the essential physical aspects, the difficulties that arise, and the methods used to overcome them, will now be described in detail.

### 2.2 Details

#### 2.2.1 Multiple Simultaneous Depositions

Our method is devoted to simulating growth on extended surfaces. All relevant computations (MD and tfMC runs) can be performed in a parallel computing environment. Therefore, the spatial extent of the studied systems is limited mainly by the available computing resources and may range from tens to even hundreds of nanometers when high-performance computing resources are available.

The fact that the simulated system has a significant extent in both in-plane directions can be exploited to increase the overall efficiency of the method. This can be done by simultaneously simulating the deposition of multiple incident atoms (and the following relaxation). In our approach *N*_mult_ atoms are deposited in each cycle. The advantage of such an approach is that it reduces the total computational effort by roughly *N*_mult_ times compared to a scenario in which atoms are deposited one by one.

Although multiple depositions are carried out within a single MD run, they can still be considered as independent events. The main reason is the large distance separating the deposition points. The method used for generating the incident atoms guarantees that they will not interact with each other on their trajectories to the surface, making the simulated process a sequence of independent single-atom depositions.

Within the implemented algorithm, the above is ensured by a suitable choice of the initial positions and velocities of the incident atoms. The generated parameters are checked for possible collisions by calculating straight-line trajectories of the incident atoms. If it is found that some incident atom will collide with another, the parameters of this atom are regenerated until a set guaranteeing no collisions is obtained. By collision, we mean a situation where two incident atoms approach one another to a distance less than *r*_c_, which is the cutoff radius of the interatomic potential used. We emphasize that the straight-line trajectories only serve as initial approximates for verifying if the chosen parameters of the incident atoms are correct. In the subsequent MD run, actual trajectories are calculated from the equations of motion.

#### 2.2.2 Using Realistic Distributions

**Figure 3:**
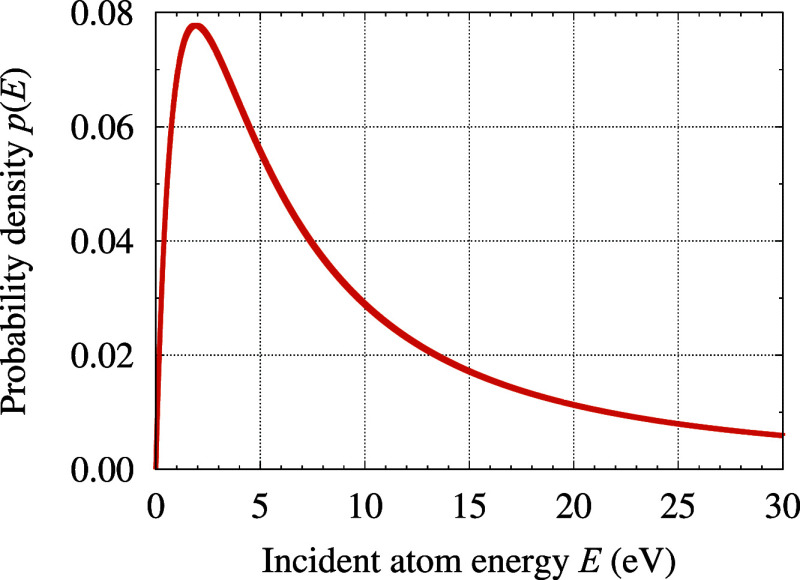
Energy distribution of the atoms ejected from the sputtering target (see eq 2). The presented example corresponds to a sputtering target made of Au, with *B* = 3.81 eV.

The realistic modeling of the deposition requires accounting for the fact that the parameters of the incident atoms, such as their energies *E* and incident angles θ, follow some distribution, often known from experiments or theory.^54−58^ In sputtering PVD techniques, the energies of the atoms ejected from the sputtering target can be described with the Thompson distribution^15,22,23,54,57,58^
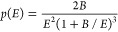
2Here, *p*(*E*) is the probability density that an atom ejected from the sputtering target will have an energy *E*, and *B* represents the cohesive energy of the sputtering target. The considered distribution (cf. Figure 3) has a specific shape with a maximum of 8/(27*B*) located at *E* = *B*/2, and a tail decaying to zero.

Equation 2 describes a situation in which the sputtering gas ions are accelerated to high energies (several hundred eV). In such a case, the energy distribution of atoms ejected from the sputtering target can be described with eq 2.

Our approach assumes that the energy distribution of the incident atoms arriving at the substrate also follows eq 2. This corresponds to assuming that on their paths to the substrate the ejected atoms do not collide/exchange energy with sputtering gas particles. Our simulation, therefore, can be thought of as corresponding to a high-vacuum regime, where the pressure of the sputtering gas is very low (for an example of how the presence of the sputtering gas can be taken into account, see, e.g., refs (15,55, and 57)). Typically, the sputtering gas moderates the energy distribution *p*(*E*), “condensing” it around lower energies. We decided to use the unmoderated distribution because it is more difficult to handle in a simulation.

This difficulty stems from the fact that the MD method uses numerical integration to find the trajectories of atoms. Accurate numerical integration requires using a sufficiently small time step, which might be extremely short for high-energy atoms. Performing constant time step integration with a time step adjusted to the fastest incident atom is not a good solution. It may lead to a situation where finding the trajectory of the slowest incident atom requires an extremely long simulation. This may result in a dramatic waste of computing power.

The above problem can be circumvented with the variable time step integration technique, which automatically adapts the time step to the fastest atom present in the MD simulation. We applied such a method (details will be given later). However, we found that this does not solve all related problems, and additional simplifications must be introduced for the simulation to be computationally efficient.

Due to the presence of the substrate and the growing TF (both are subjected to thermal conditions), the employed time step cannot be increased above a certain threshold (typically in the order of fs). With such a time step the incident atoms with very low energy cannot be efficiently handled. These atoms may move with arbitrarily low velocities (note that the left side of the distribution (2) extends to *E* = 0), and determining their complete trajectories would necessitate impractically long simulation times.

Furthermore, the behavior of the very high energy incident atoms (note that distribution (2) extends to infinite energies) is problematic to model too. The underlying reason is that the empirical potentials that describe interatomic interactions often cannot deal with situations where two atoms get very close. Such situations can be expected in high-energy collisions. Unphysical behavior can be expected in such cases or even singularities in forces or potential.

The above means that accounting for the variability of *E* is difficult in a computationally efficient and physically sound simulation. For this reason, we decided to truncate the energy distribution (2), taking
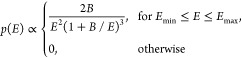
3This, of course, eliminates incident atoms on both extremes of energy. Because of their relatively low occurrence, this can be expected to have a negligible influence on the growth of the TF. The threshold energies (*E*_min_ and *E*_max_) can be chosen so that the neglected parts of *p*(*E*) correspond to a low fraction of the incident atoms.

In our method, the incident atoms are initially positioned far from the substrate’s surface and far from each other. Therefore, they do not interact with the surface or with each other. Consequently, their energies are controlled through their initial velocities. These velocities are generated randomly, such that the distribution of kinetic energies follows the truncated distribution defined by eq 3.

It is known that the geometry of the PVD experimental setup may influence the characteristics of the grown TF. The underlying reason is that the parameters, such as the sizes of the sputtering target and substrate, and their separation, influence the distribution of the incident angles θ, at which the incident atoms arrive at the substrate. In the developed method we also accounted for this effect.

The method used for generating the initial velocities ensures that the incident angles follow the assumed distribution. We took the cosine distribution^57,59,60^
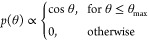
4truncating it at θ_max_, which represents the maximum incident angle, a parameter related to the geometry of the experimental setup.

The initial positions of the incident atoms are also generated randomly, with the uniform distribution used for all three Cartesian directions. The incident atoms are positioned within a cuboid-shaped region with height *z*_h_, separated along the *z* direction by *z*_d_ from the top layer of the growing TF. In the adopted approach, the generation region moves because its location is determined on the fly by searching in the growing TF for the atom with the maximum *z* component of the position.

#### 2.2.3 Simulating Collisions and Fast Relaxation

The generated incident atoms are added to the system, and their collisions are simulated in the MD run. In this run, the initial positions of the remaining nonincident atoms, i.e. substrate atoms and atoms deposited earlier, are taken from the preceding run. For the first deposition cycle, this is the substrate equilibration MD run. In subsequent cycles, it is the tfMC run from the previous deposition cycle.

Similarly, the initial velocities of all nonincident atoms are taken from the previous MD run. This is the substrate equilibration MD run (for the first deposition cycle) or the MD run from the previous deposition cycle (for subsequent deposition cycles). Such a choice is better than, e.g., using new, randomly generated velocities, because it speeds up equilibration at the very beginning of the MD run. This equilibration is explicitly accounted for, as each MD run starts with a short equilibration period (consisting of *N*_init_^MD^ steps, performed with a constant time step) when the incident atoms are temporarily fixed, so they do not collide with the not yet equilibrated surface.

After this equilibration, the incident atoms become mobile, and the actual simulation of their collisions with the surface begins. In this stage, the equations of motion are integrated using variable time step integration (details will be given later). This ensures the calculated trajectories are accurate, even for high-energy incident atoms.

The generated incident atoms cover a wide range of initial velocities and positions. This causes a significant spread in the times required for their reaching the surface. Our protocol accounts for this, ensuring that each MD run is sufficiently long. The calculated straight-line trajectories of the incident atoms are used to estimate the time required to reach the surface by all incident atoms. This information is passed to the MD run, whose length is adjusted accordingly, accounting for the variable time step used. The above explains why the individual MD runs differ in length in our protocol.

The MD run does not stop immediately after all collisions have occurred, and the system is simulated for some additional time (*N*_final_^MD^ steps, with a constant time step). This ensures that fast relaxation occurring after the collisions will also be accounted for.

The incident atoms carry energy, which is transferred to the surface through collisions. This transfer of energy may cause a heating of the surface. In order to simulate real conditions where the bulk of the substrate moderates the temperature of the TF being deposited, we have applied temperature control. In all MD runs, the system’s temperature is kept constant at a specified target temperature *T*_dep_ by applying the Langevin thermostat^61^ to all atoms except the immobile and incident atoms. We do not thermostat—as is often done^7,9,11,13,15,19−21,23−25^—a smaller group of deeper-located substrate atoms—our goal is to maintain consistency between MD and tfMC runs. The latter (see below) naturally sample the canonical (*NVT*) ensemble, keeping the temperature constant in the entire system.

#### 2.2.4 Simulating Slow Relaxation

The structure obtained from the MD run is subsequently subjected to an tfMC run and simulated for a given number of tfMC steps (*N*_relax_^tfMC^), the same in each cycle. In each tfMC step, all atoms except the immobile atoms are displaced according to the tfMC random moves (for details on their construction, see ref (30), in particular eqs 9–11 therein). In these moves, the maximum displacement of atom *i* (which is element-specific) is taken as^31^
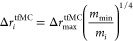
5Here, *m*_*i*_ denotes the mass of atom *i*, while Δ*r*_max_^tfMC^ represents the maximum displacement of the lightest atom (with mass *m*_min_). In all tfMC runs we use the same value of Δ*r*_max_^tfMC^.

Because the tfMC algorithm samples the *NVT* ensemble, no additional thermostatting is required. In all tfMC runs, the sampling temperature is the same as the target temperature of the MD runs (*T*_dep_), ensuring the simulated deposition is carried out under isothermal conditions.

The length of the final tfMC run (*N*_final_^tfMC^) can be chosen arbitrarily. However, a reasonable choice is to perform at least *N*_final_^tfMC^ = *N*_cycl_ × *N*_relax_^tfMC^ steps, extending the length of the simulation by the total number of tfMC steps taken in the main loop. This makes it very likely that in the TF produced, the surface will be relaxed to a degree comparable to that of deeper layers.

#### 2.2.5 Removing Vapor Atoms

Occasionally, due to a collision, an incident atom will be reflected, and/or some surface atoms will be resputtered. Although the MD method used for simulating collisions reproduces such events, it does not allow efficient simulating of the entire escape of the reflected and resputtered atoms, especially those with very low kinetic energies.

Because of that, the approach as described above might lead to an excess accumulation of reflected and resputtered atoms above the surface, and we observed this phenomenon in numerical experiments. Such formation of a vapor phase is also observed in reality, but its density is significantly lower. Therefore, the observed accumulation of excess vapor should be considered an artifact of the simulation method used, particularly the short times used in the MD runs, and the “freezing” of translational motions in the tfMC runs. This excess vapor may affect deposition, as its particles constitute obstacles for the incident atoms.

To solve the above problem the reflected and resputtered atoms are removed after each MD and each tfMC run (see Figure 2a). The atomic configuration obtained from the run is checked for the possible presence of atoms not bonded to the substrate, forming free atoms or free clusters. These vapor atoms are removed from the simulation. We use a simple (distance-based) criterion to identify such atoms: two atoms are said to be bonded if the distance between them is less than the specified bonding cutoff *r*_b_. The atom is considered a vapor atom if no continuous chain of bonds connects it to the substrate. We thus reformulate the considered problem as finding all disconnected subgraphs of a graph, with atoms being the graph vertices, bonds being the graph edges, and vapor particles corresponding to smaller subgraphs, which are disconnected from the largest subgraph, constituted by the substrate and the deposited TF. Our implementation solves this problem with the breadth-first search method. The identification of bonds is carried out using the linked-cell list method.^62^

### 2.3 Implementation

The approach described above was implemented on top of LAMMPS,^63^ which was used in all MD and tfMC runs in this work. Our implementation primarily consists of LAMMPS scripts that invoke its various commands. Relying on LAMMPS as a simulation engine offers several advantages. First, LAMMPS provides a wide choice of particle and interaction models. This opens the possibility to study the deposition of different films on selected substrates. Second, LAMMPS is a parallel code, allowing each MD and tfMC run in our protocol to utilize multiple CPU cores, which facilitates the efficient study of large systems.

The aspects of our approach which could not be achieved through the functionalities of LAMMPS (see color coding in Figure 2a) were implemented in overseer—a separate program, written in C++. This program is called before MD and tfMC runs. It is responsible for aspects such as the generation of the incident atoms, passing between runs the positions and velocities of other (nonincident) atoms, removing the vapor atoms, and determining and passing to LAMMPS all the required parameters such as the lengths of the MD runs of deposition. In short, the overseer prepares the initial atomic configurations required for the run by processing the configuration obtained from the previous run. It also collects measures which are of particular interest, such as the statistics describing the results of the individual depositions (the numbers of deposited, reflected and resputtered atoms, histograms of energies and incident angles), and the quantities describing the thermodynamic state of the simulated system (its temperature, energy, and others).

The proposed approach to simulating deposition is very general. In the presented description, we have limited ourselves to the growth achieved through the PVD technique, which is why we assumed distributions (3) and (4). However, other energy and incident angle distributions can be implemented with little effort, opening the possibility of modeling other deposition techniques, like chemical vapor deposition. As for now, the developed implementation considers the deposition of a monatomic film on a monatomic substrate. However, the extension to polyatomic materials is possible and straightforward.

The developed implementation is available from the corresponding author, upon reasonable request. It will be made publicly available in the near future.

## 3 Simulations and Analyses

### 3.1 Simulation Details

To demonstrate the practicability of the proposed method, we used it to study the deposition of a 3 nm thick Au film on crystalline Si, with a diamond cubic (dc) structure and (001) surface orientation.

For describing interactions we used a modified embedded atom method (MEAM) potential proposed by Ryu and Cai,^64^ and developed specifically for the Au–Si system. This potential was constructed^65^ from two MEAM potentials available for monatomic systems (Si^66^ and Au^67^) by fitting the parameters describing the interactions of unlike atoms (Au–Si). As shown in ref (64), the proposed parametrization correctly reproduces the essential properties (lattice parameters, bulk modulus, elastic constants, and cohesive energy) of pure fcc Au and dc Si systems, and of the hypothetical Au–Si alloy (with the rock salt structure). It also correctly reproduces the binary bulk phase diagram of the Au_1–*x*_Si_*x*_ system, providing—among others—the eutectic temperature (*T*_e_ = 629 K) and eutectic composition (*x*_e_ = 0.234), which agree well with experiment (634 K and 0.195, respectively). This was the main reason for choosing this potential.

It should be noted that the investigated Au–Si system exhibits a mixed bonding character, combining covalent (crystalline Si) and metallic (deposited Au TF) components. Therefore, to accurately describe the interactions, the MEAM formalism must be employed. The MEAM approach is an extension of the embedded atom method,^68,69^ with the key enhancement being the inclusion of angular (nonspherically symmetric) contributions to the electron density.^66^ This enables the MEAM model to capture directional bonding, which is essential for reliable treatment of covalent materials and also beneficial for improving the description of transition metals.

The Si substrate initially had dimensions 21.8 nm × 21.8 nm × 2.7 nm (40 × 40 × 5 repetitions of the 8-atom dc unit cell) and contained 64,000 Si atoms. Out of these, 12,800 atoms belonged to the immobile group, which encompassed four (001) crystal planes. The lattice parameter of the Si substrate was taken as *a*_Si_ = 5.453 Å, which is the equilibrium value determined for the assumed temperature *T*_dep_ = 300 K and zero pressure.

In generating the incident atoms we assumed *B* = 3.81 eV, which is the cohesive energy of fcc Au.^70^ The threshold energies were taken as *E*_min_ = 0.5 and *E*_max_ = 100 eV, neglecting 1.3% and 7.2% of the incident atoms with the lowest and highest energies, respectively. The threshold angle was taken as θ_max_ = 25°, corresponding to a typical experimental setup. The incident atoms were generated in a region with a height of *z*_h_ = 50 Å, located *z*_d_ = 10 Å above the growing film.

For the identification of collisions we used *r*_c_ = 8.4 Å, which is the cutoff radius of the MEAM potential used. In identifying vapor atoms, we used *r*_b_ = 4.2 Å, corresponding to a midpoint between the second and third coordination shell in dc Si. This value is higher than the lattice parameter of fcc Au (*a*_Au_ = 4.095 Å).

The MD runs corresponding to substrate equilibration, initializations and finalizations of depositions used a constant time step of Δ*t*^MD^ = 0.5 fs. We motivate the choice of such a conservative value by the fact that the deposited TF contained many interstitial Si atoms, which oscillate with higher frequencies, requiring a shorter time step for accurate integration. In the MD runs of actual deposition, the variable time step integration was effected through the fix dt/reset command of LAMMPS. The time step was adjusted every 20 timesteps and the method used for its adjustment guaranteed that in a single time step, none of the atoms moved by more than 0.005 Å and that the kinetic energy of any atom did not change by more than 1 eV. In all tfMC runs, the Δ*r*_max_^tfMC^ parameter was set to 0.10 Å, which constitutes ≈4% of the shortest atom–atom distance (Si–Si) and, therefore, is a conservative value.

After equilibrating the generated substrate (the corresponding MD run used *N*_equil_^MD^ = 20,000 steps), we deposited 80,000 Au atoms by running *N*_cycl_ = 2500 cycles, and depositing *N*_mult_ = 32 atoms in each cycle. A single MD run consisted of approximately 58,000 steps (with *N*_init_^MD^ = 4000, *N*_deposit_^MD^ ≈ 44,000 on average, and *N*_final_^MD^ = 10,000). In each tfMC run we used a comparable number of steps, taking *N*_relax_^tfMC^ = 50,000. The final tfMC run consisted of a further *N*_final_^tfMC^ = *N*_cycl_ × *N*_relax_^tfMC^ = 2500 × 50,000 steps. Consequently, the entire simulation consisted of approximately 400 M steps. It required 75 days to complete when run continuously on 32 cluster nodes, with each node equipped with two 12-core Intel Haswell 2.3 GHz processors. To underline the effort involved in performing it, we note that the total computational effort of this simulation was ≈1.4 M core hours. The results of this simulation are presented in Section 4.

We have also investigated the benefits of applying the tfMC method to extend the time scale. For this purpose, we performed three sets of additional simulations.

In the first set of simulations, we observed the long-time evolution of the TF obtained from the tfMC + MD simulation. For this purpose, configurations corresponding to various stages of the growth were taken, and their further evolution was simulated using either tfMC or MD. By comparing descriptors describing the observed evolution we determined ⟨Δ*t*^tfMC^⟩, quantifying the speed-up that the tfMC method offers compared to MD. Details and results are presented in Section 5.1.

The second set were additional simulations of growth. They were similar to the deposition simulation described above, as most of their parameters were identical. The only difference was that the long-time relaxation was either (i) not simulated or (ii) simulated with the MD method. In the first case, the tfMC runs were not performed (by setting *N*_relax_^tfMC^ = 0), while in the latter case, all the tfMC runs were replaced with MD runs of the same length (by setting *N*_relax_^tfMC^ = 0 and increasing *N*_final_^MD^ to 60,000).

These two additional simulations can be viewed as scenarios in which the long-time relaxation is either not simulated, or is simulated, but with MD. The three considered approaches will be denoted as MD + tfMC (original method), MD, and MD + MD. The additional simulations allowed us to assess the practical benefits of using the tfMC method. This was achieved by comparing the TFs produced from the three considered simulation approaches, particularly their energetics, structure, and morphology. The results of this comparison are presented in Section 5.2.

In the third set of simulations, we observed how the choice of the Δ*r*_max_^tfMC^ parameter influenced the obtained picture. For this purpose, we performed a series of tfMC + MD simulations, which differed in Δ*r*_max_^tfMC^. In addition to the original 0.10 Å, the following values were considered (all in Å): 0.05, 0.15, 0.20, and 0.25. These simulations allowed us to check which Δ*r*_max_^tfMC^ values are safe and what other benefits the tfMC technique offers, if higher (but theoretically still acceptable) Δ*r*_max_^tfMC^ values are used. The results of this study are presented in the Supporting Information.

### 3.2 Methods

We analyzed the TFs obtained from the simulations from a number of perspectives: energetic, structural, and morphological, among others. For this purpose, we used a variety of measures and analysis techniques, which will now be introduced.

The energy analysis was carried out based on the concept of the formation energy *E*_form_^TF^, which measures the energy of the “product” (i.e., the TF obtained from the deposition process) relative to the “reactants” used. In the considered case, the “reactants” were the perfect Si and Au crystals, which would be used as the substrate and the sputtering target in the experiment. Therefore, the formation energy *E*_form_^TF^ was calculated from the equation
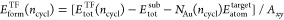
6Here, *E*_tot_^TF^ represents the total energy of the growing TF. Its value depends on the deposition cycle. To specify it, we will use the number of deposition cycles already taken (*n*_cycl_) throughout this work.

In eq 6*E*_tot_^sub^ represents the total energy of the substrate. Its value was obtained from the substrate equilibration run.

The product of *N*_Au_ and *E*_atom_^target^ represents the energy of the sputtering target, that is, the energy that *N*_Au_ gold atoms would have if they were embedded in the sputtering target. Here, *N*_Au_ denotes the number of the deposited Au atoms (its value depends on *n*_cycl_), while *E*_atom_^target^ represents the energy of a single Au atom in the sputtering target. For simplicity, we assumed that all deposited Au atoms were obtained from the bulk of the sputtering target. Therefore, the value of *E*_atom_^target^ was obtained from a separate MD run, which simulated a bulk Au monocrystal with a perfect fcc structure. This simulation assumed *a*_Au_ = 4.095 Å, the equilibrium lattice parameter for *T* = 300 K (same as in the simulated deposition) and zero pressure.

*A*_*xy*_ appearing in the denominator of eq 6 represents the *xy* cross-sectional area of the simulation box. Its presence gives *E*_form_^TF^ the meaning of a surface specific energy, expressing the minimum energy required to form a unit area (1 nm^2^) of the TF with a given thickness (given implicitly by *n*_cycl_). Throughout this work, the TF’s thickness will also be expressed by specifying the number of perfect Au(111) crystal planes with area *A*_*xy*_ containing the same number of Au atoms. The number of monolayers (MLs) considered here was calculated from the formula
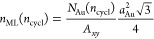
7

To characterize the structural changes occurring in the TF and substrate, we used two structure analysis methods. The polyhedral template matching^71^ (PTM) method was used to characterize the local structure around the Au atoms, distinguishing the following packing types: fcc, hcp, and bcc. Au atoms which did not display any of these packings were classified as other. This group also contains all Au atoms located on the surface of the TF because the PTM method cannot identify crystal surfaces. In the PTM analysis, the cutoff for root-mean-square deviation was set to 0.15.

The local structure around the Si atoms was analyzed using a method proposed by Maras et al.,^72^ which relies on common neighbor analysis^73,74^ and will be termed identify diamond structure (IDS). Based on it, each Si atom was classified as possessing either perfect-dc, defective-dc, or nondc structure. Here, by perfect-dc atoms we mean atoms that are positioned on the dc lattice, and have a complete set of first and second neighbors, all of which are also positioned on the dc lattice sites. By defective-dc atoms, we mean atoms that are positioned on a dc lattice but either have an incomplete set of neighbors or have—among their first and second neighbors—atoms which are not positioned on the dc lattice sites. By nondc Si atoms, we mean atoms that do not belong to either of these two groups.

Prior to performing structure analysis, the configurations of TF were energy minimized in order to remove the influence of thermal vibrations. This facilitated structural analysis and enabled a direct comparison of results obtained from the three considered approaches (MD + tfMC, MD + MD, and MD). The values of measures incorporating thermal effects (such as *E*_tot_^TF^ appearing in eq 6, the ρ(*z*) density profiles described below) were obtained by time-averaging the data obtained from the MD runs corresponding to finalizations of depositions. This also ensured the comparability of results between approaches.

To characterize the morphology of the TF we used the so-called density profile ρ(*z*), which informs how the atomic number density (expressed in atoms/nm^3^) changes along the out-of-plane direction (*z*). The ρ(*z*) profiles were calculated by dividing the simulation box into thin slabs with a width of Δ*z* = 0.02 nm and counting the number of atoms residing in each slab. Separate profiles were determined for Si and Au atoms. They were further structure-decomposed based on the IDS and PTM methods, respectively.

We also used other analysis methods, including the dislocation extraction algorithm (DXA) proposed by Stukowski,^75,76^ implemented in OVITO,^77^ a program that we used for performing structural analyses (PTM, IDS and DXA) and for preparing visualizations.

## 4 Example Application

### 4.1 Characteristics of the Growth

**Figure 4:**
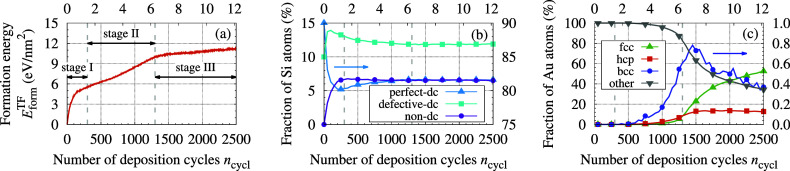
Energy and structure of the growing TF, as predicted from the MD + tfMC simulation. Panel (a) shows the evolution of the formation energy *E*_form_^TF^ (see eq 6). Panels (b,c) present the results of structural analysis carried out with IDS (b) and PTM (c) methods, for Si (b) and Au (c) atoms, respectively. In all panels, the dashed vertical lines delineate the three identified stages. For convenience, the top horizontal axis presents the thickness of TF, expressed as the number of monolayers *n*_ML_ (see eq 7). As indicated with an arrow, in panel (b), the data corresponding to the perfect-dc class uses the right axis. The same holds for panel (c) and the bcc class.

**Figure 5:**
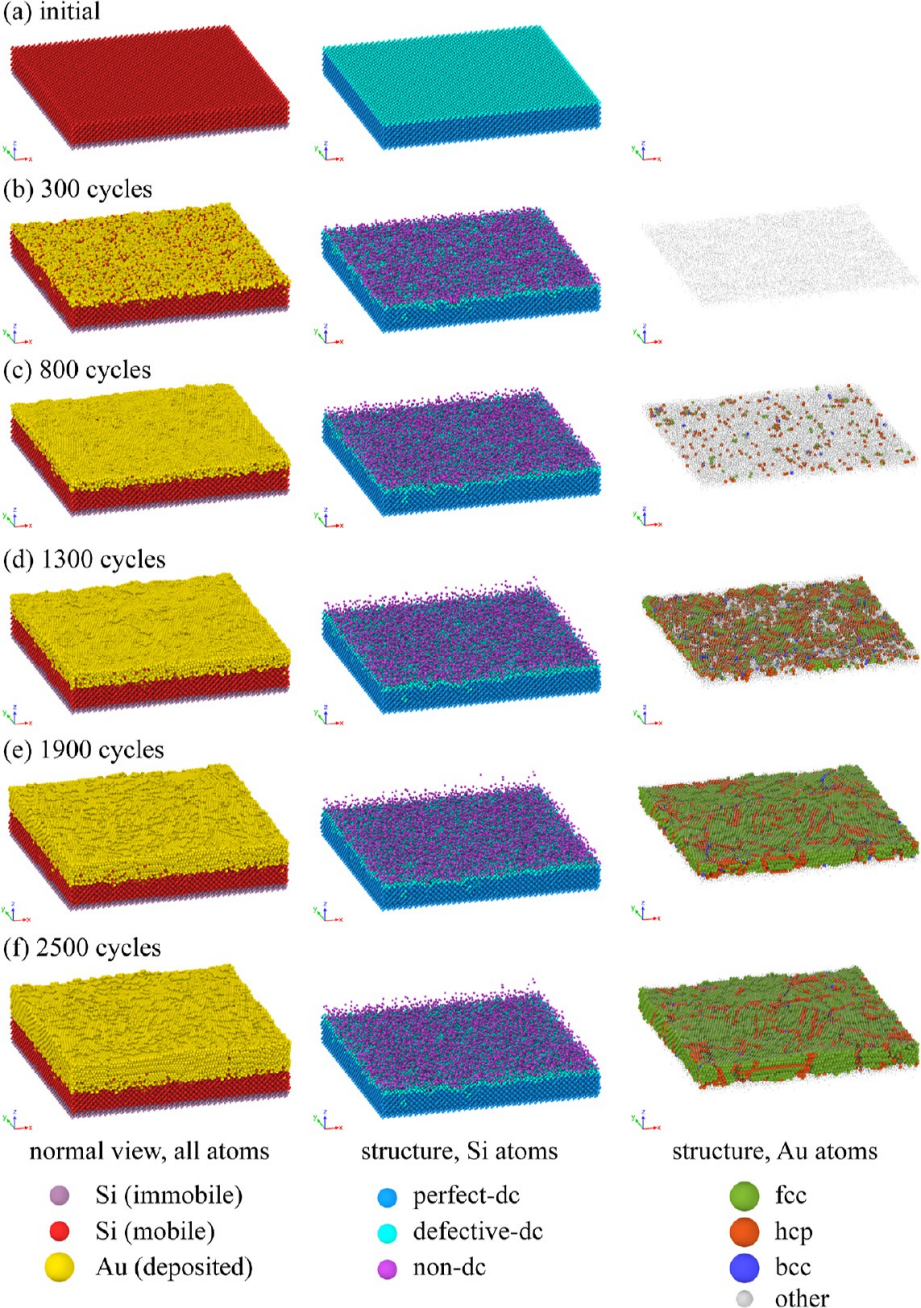
Visualizations of the growing TF. Successive rows correspond to stages of growth. The left column presents the entire system, while the middle and right columns show, respectively, only the Si or Au atoms, colored according to their local structure. In the interest of readability, unclassified Au atoms are represented with smaller spheres.

We start the presentation of results with a description of the growth process. Figure 4a shows how the formation energy of the TF evolved in the MD + tfMC simulation. Initially, the formation energy *E*_form_^TF^ was increasing abruptly. A subsequent decrease in the slope of *E*_form_^TF^ vs *n*_cycl_, which occurred at *n*_cycl_ ≈ 100–200, indicates that the character of the growth changed between these cycles. This behavior, where the increase in the formation energy was moderate, lasted until ≈1300 cycles. After this point, another drop in the slope was observed, indicating another change in the character of the growth. We can thus distinguish three distinct stages in the growth process: I, II, and III. Their character can be better understood with the aid of structural analysis (Figure 4b,c) and visualization (Figure 5).

The stage I (up to ≈300 cycles) was characterized by visible degradation of the substrate surface caused by the arriving incident Au atoms, particularly the highly energetic ones. By penetrating the Si substrate, these atoms introduced numerous defects in its top layer, releasing some Si atoms from the substrate, which, consequently, became rough. The released Si atoms and the deposited Au atoms formed a mixed Au–Si layer, constituting an interface between the Si substrate and the Au TF being deposited. In stage I, many implantations of Au atoms also occurred. These were often deep, reaching 1 nm below the surface of the initial substrate.

Figure 5a,b illustrate the effects identified above, showing the simulated system after 300 deposition cycles. The described effects are also well visible in Figure 4b, which shows a significant increase in the fraction of Si atoms classified as defective-dc and nondc. After 300 cycles, these fractions reached 13.2% and 6.6%, while initially they were 10% (defective-dc atoms forming surface) and 0%, respectively. Consequently, in stage I, the fraction of Si atoms classified as perfect-dc changed from 90% to 80.2%, decreasing by 6203 atoms. This number corresponds to destroying almost two full Si(001) dc crystal planes.

**Figure 6:**
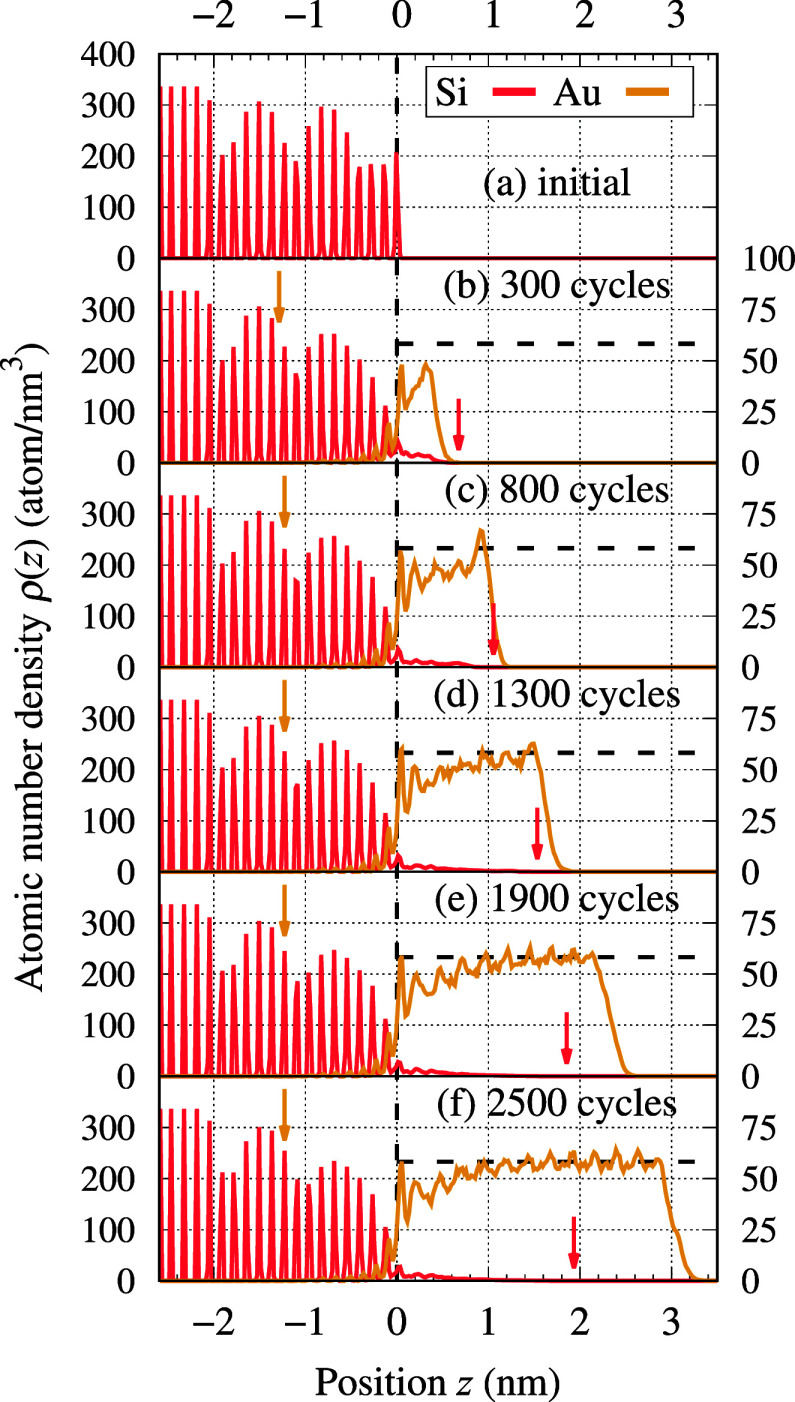
Density profiles of the growing TF. The state of TF is characterized at the same stages of growth as those shown in Figure 5. For convenience, the horizontal axis was shifted such that *z* = 0 corresponds to the initial location of the Si substrate’s surface (indicated with a vertical dashed line). Two vertical axes should be used for reading data for Si (left) and Au (right). Both express ρ(*z*) in atom/nm^3^. The color arrows indicate the location of the Si atom that diffused the most into the Au TF (red) and the deepest implanted Au atom (gold). The horizontal dashed lines depict the density of bulk Au.

In stage II, spanning cycles 300–1300, further transport of Si atoms released from the substrate was observed (see Figure 6, panels (b,d)). These atoms continuously diffused into the Au TF being deposited, with high-energy collisions driving this process. As seen in Figure 4b, between cycles 300–1300, the fraction of Si atoms classified as perfect-dc increased by nearly 1.5 percentage point (p.p.). This shows that during stage II self-healing of the Si substrate took place, leading to a reconstruction of crystal structure around many Si atoms, which belonged to the defective-dc class after the initial depositions.

Ordering of the deposited Au TF also started in stage II. Initially, it occurred through the development of short-range order, which manifested in an increase of the fractions of Au atoms, which—temporarily—displayed hcp and fcc packings (see Figure 4c, *n*_cycl_ ≈700 and later cycles). As the deposition proceeded, the amount of Au atoms with hcp- and fcc-like nearest neighborhoods grew constantly. This—at the end of stage II—led to the formation of larger crystalline-like agglomerates.

Once stable nuclei of the Au fcc-phase were formed at the end of stage II, the further growth of the TF proceeded in an ordered fashion through the growth of the formed Au nanocrystallites. Such behavior was observed in stage III, which started at ≈1300 cycles. From this point on, further deposition of Au atoms led to a constant growth of the formed nanocrystallites in the out-of-plane (+*z*) direction (see Figure 5e,f). It is worth highlighting that for up to 1300 cycles (the beginning of stage III), the fraction of hcp-like Au atoms was higher than that of Au atoms with fcc packing. This observation suggests that the ordered growth of TF began when the fcc phase started dominating.

Au atoms with a bcc-like structure were also observed in the growing TF. However, their relative occurrence was low and did not exceed 0.78%, the maximum observed at 1450 cycles. This fraction later decreased to 0.37% at 2500 cycles.

The formation of nanocrystallites also influenced the mixed Au–Si interface layer, which became partially ordered in stage III, as a consequence of crystallization proceeding in the downward (−*z*) direction. This can be clearly seen in the density profiles in Figure 6, panels (d–f), where the Au peaks corresponding to the interface region become sharper with time.

Significant diffusion of Si atoms was observed over the entire deposition process. The Si atoms released from the substrate in stage I continuously diffused into the growing TF. At the end of the deposition process (i.e., after *n*_cycl_ = 2500 cycles), the Si atom that diffused the most was found as deep as 2 nm within the bulk of the Au TF (see Figure 6f). A more detailed description of Si diffusivity (e.g., determining the corresponding diffusion coefficient) is hindered by insufficient sampling. The unknown timespan of the tfMC runs additionally makes a quantitative description of the diffusion of Au atoms problematic. However, we note that surface diffusion emerged as the dominant mechanism in the case of Au atoms, manifesting once Au nanocrystallites were formed.

Regarding the surface morphology and the growth character, the Au TF being deposited almost from the beginning covered the entire substrate and did not form isolated islands, which are typical of the Volmer–Weber growth mode. This observation suggests that the considered growth should be classified as a layer-by-layer growth, i.e. the Frank-van der Merwe mode.^78^ However, the observed growth was not strictly two-dimensional, as the TF formed was at all times characterized by significant roughness, reaching 1 nm. The picture obtained from the MD + tfMC simulation is consistent with recent experiments,^79^ which also observed that the PVD of Au on the crystalline Si proceeds in a fashion close to the Frank-van der Merwe mode, and also produces TFs with considerable roughness (ref (79) reported ≈3 nm roughness for a 10 nm thick TF).

Regarding the results of the individual depositions, the sticking coefficient was very high. Out of 80,000 incident Au atoms, only 521 atoms (i.e., 0.65%) failed to deposit. Only one Si atom was sputtered from the Si substrate.

Animations of the MD + tfMC simulation deposited in ref (80) (accessible through the Bridge of Knowledge portal of the Gdańsk University of Technology) present the observations made in this section in more detail.

### 4.2 Postdeposition Relaxation

Following deposition, the obtained TF was further evolved in the final tfMC run, which can formally be classified as another stage of growth (stage IV). Performing it allowed to account for various relaxation processes. As seen in Table 1, these processes caused visible structural changes, leading to a considerable (0.5 eV/nm^2^) decrease in the formation energy of the TF. The most pronounced structural change was an increase in the crystallinity of the produced Au TF, which manifested in a 1.6 p.p. increase in the fraction of Au atoms displaying fcc packing. This change was accompanied by a 1.2 p.p. decrease in the fraction of Au atoms belonging to the other class. Although significant, these numbers do not fully reflect the scale of the structural changes that occurred.

**Table 1:** Measures Characterizing the TF Obtained from MD + tfMC Simulation^a^

	TF state	
	as-deposited	relaxed
formation energy (eV/nm^2^)	11.23	10.76
local structures in the entire system (%)		
Si atoms		
perfect-dc	81.61	81.61
defective-dc	11.89	11.84
nondc	6.50	6.55
Au atoms		
fcc	52.40	54.02
hcp	12.74	12.34
bcc	0.37	0.33
other	34.49	33.31

a Two states of the TF are compared: immediately after the deposition process (“as-deposited”) and after the final tfMC run (“relaxed”). The first row presents the formation energy *E*_form_^TF^ (see eq 6). The remaining rows present the structural analysis carried out with the IDS and PTM techniques. Values correspond to fractions (expressed in %) of atoms of a given type (Si or Au) displaying the given packing type.

To better illustrate these changes, we performed a detailed analysis, investigating structural transformations with atomic resolution. In particular, for each atom in the system we checked if its packing type, as obtained from IDS or PTM, had changed throughout the final tfMC run. This was done by comparing packing types determined at the end of the deposition and the end of the final tfMC run, i.e., the same configurations as those reported in Table 1.

As seen in Table 2 (see rows described as “all transformations”), more than 9% of all Au atoms and about 2% of all Si atoms changed their packing type. This clearly shows that the fraction of the system’s atoms actively participating in the observed postdeposition relaxation was considerably larger than what would be suggested by a pairwise comparison of the columns of Table 1. Here, summing the absolute changes would inform us that only 3.2% of Au atoms and 0.1% of Si atoms had transformed, which is misleading, because, for example, the effects of the concurrent transformations, such as other → fcc and fcc → other, effectively cancel out in this picture.

**Table 2:** Structural Transformations Occurring During Postdeposition Relaxation^a^

transformation type	number of transformations	
Si (63,999 atoms)		
defective-dc → perfect-dc	434	(0.68%)
perfect-dc → defective-dc	434	(0.68%)
defective-dc → nondc	191	(0.30%)
nondc → defective-dc	158	(0.25%)
nondc → perfect-dc	2	(≈0%)
perfect-dc → nondc	0	(0%)
all transformations	1219	(1.90%)

transformation type	number of transformations	
Au (79,479 atoms)		
other → fcc	1745	(2.20%)
hcp → fcc	1273	(1.60%)
other → hcp	1131	(1.42%)
fcc → other	1007	(1.27%)
hcp → other	909	(1.14%)
fcc → hcp	737	(0.93%)
bcc → hcp	85	(0.11%)
bcc → other	83	(0.10%)
hcp → bcc	79	(0.10%)
bcc → fcc	74	(0.09%)
other → bcc	65	(0.08%)
fcc → bcc	64	(0.08%)
all transformations	7252	(9.12%)

a The consecutive rows present the number of transformations of various types, starting from the most common. The number in the bracket informs to what fraction of atoms of the given type (Si or Au), the given number of transformations corresponds. The total number of transformations is also given (in the last rows).

In the case of Si atoms, the transformations between perfect-dc and defective-dc classes dominated, constituting ≈70% of all transformations observed for Si. The remaining 30% were predominantly transformations between defective-dc and nondc classes.

For Au, the transformations into the fcc structure (other → fcc and hcp → fcc) were the most frequent, accounting for as much as 42% of all transformations observed for Au. The number of transformations from the fcc structure (fcc → other and fcc → hcp) was, however, also significant, constituting 24% of all Au’s transformations. Equally important were the transformations between the hcp structure and the other class (hcp → other and other → hcp), accounting for 28% of all Au transformations. The remaining 6% were various transformations from and to the bcc class.

**Figure 7:**
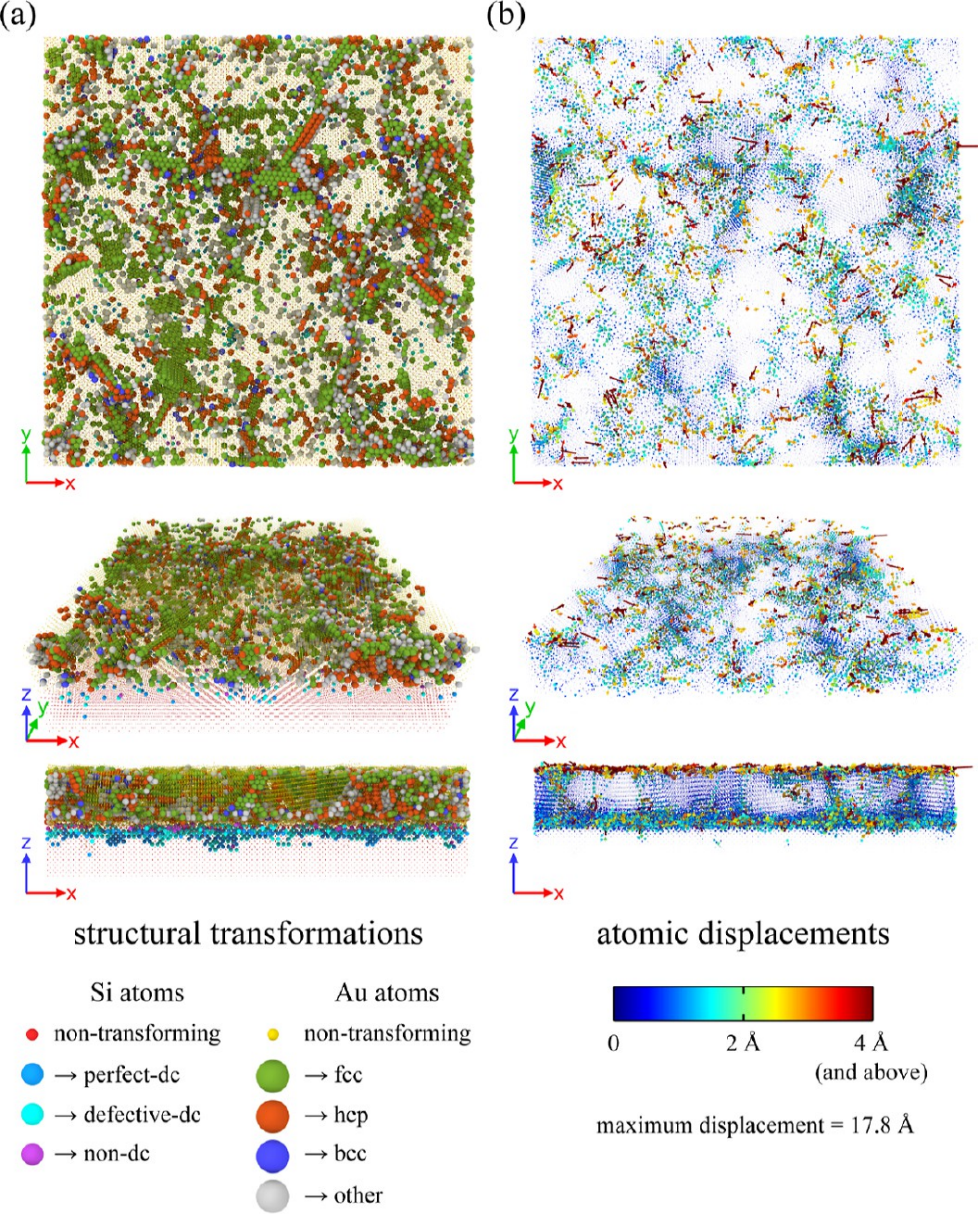
Structural transformations occurring during postdeposition relaxation. Various views (top, fly, and side) are presented. Panel (a) presents the system state after the final tfMC run. The transforming atoms were colored according to their final structure type. The nontransforming atoms are also shown, but are represented with smaller spheres. Panel (b) visualizes atomic displacements. Each atom is represented with a vector, connecting its initial and final positions in the final tfMC run. Vectors were colored according to their length (see the provided color key).

**Figure 8:**
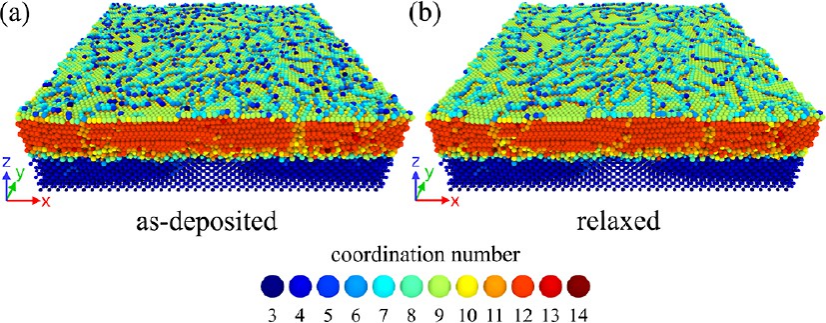
Postdeposition relaxation of the surface. The two panels present the simulated system immediately after the deposition (a), and after the final tfMC run (b). Atoms were colored according to their coordination number. A 3.5 Å cutoff was used. Due to surface diffusion, many surface Au atoms, which were initially low-coordinated, moved to step positions, increasing their coordination number. This process led to the formation of a smoother surface.

**Figure 9:**
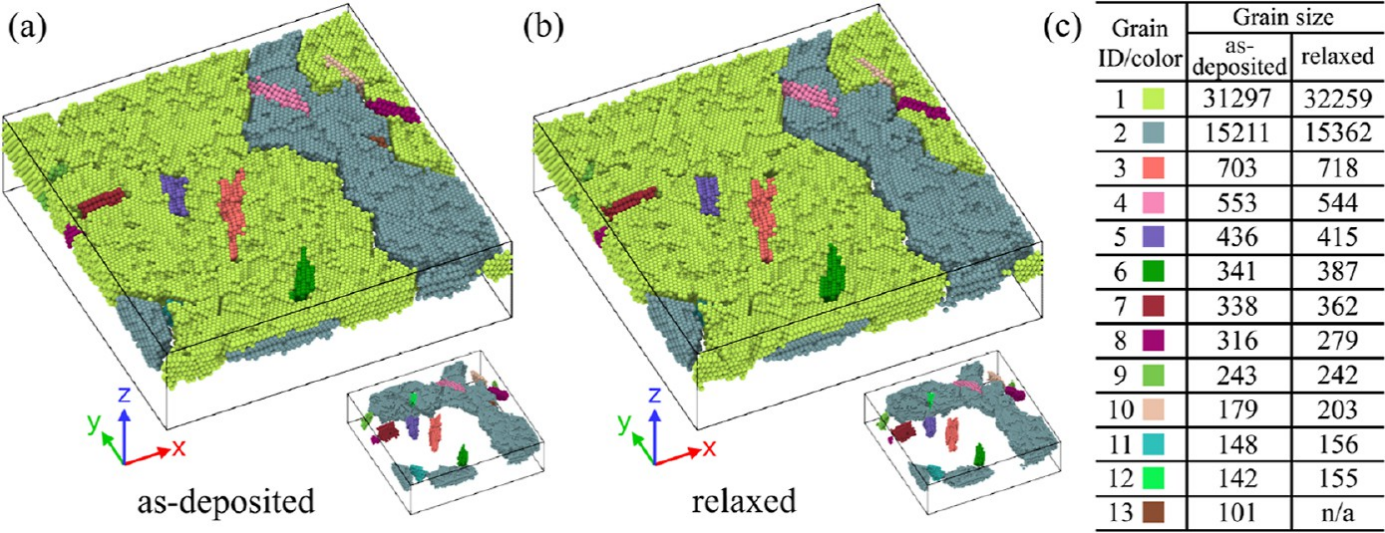
Reorganization of the grain structure occurring during postdeposition relaxation. Both panels present the grain structure, showing the simulated system immediately after the deposition (a), and after the final tfMC run (b). In both panels, Au atoms belonging to the same grain are shown with the same color. In order to enhance readability, all Si atoms and Au atoms which were classified as other or were determined as not belonging to any grain, were removed from the visualization. The table in panel (c) maps colors to grains and shows their sizes (number of atoms). Note that in the considered case, the two largest grains are partially stacked on top of each other, with grain #1 lying on top of grain #2. To clarify this, in insets below atoms of grain #1 were removed. The grains were identified with OVITO’s^77^ grain segmentation method, using the minimum spanning tree algorithm, with the minimum grain size set to 100 atoms and the merge threshold (the maximum misorientation angle) of 5°.

The diversity of the observed transformations reveals a rather complicated character of the structural changes that took place after the deposition finished. This was confirmed with a spatially resolved analysis (see Figure 7), revealing that the observed transformations occurred almost in the entire system, encompassing the surface and the bulk of the fcc Au layer, the Au–Si interface layer, and the topmost fragment of the Si substrate. In addition, the observed structural rearrangements often had a complex and many-body character, involving the transformation of structure and collective motions of many nearby atoms. These complex transformations correspond to various relaxation processes, which would also occur in reality in a similar system, i.e., the highly defective as-deposited TF. Among the observed processes we identified: surface diffusion of low-coordinated surface atoms (see Figure 8), diffusion and healing of point defects, motions of dislocations and the related stacking faults, and reorganization of grain boundaries, constituting onsets of the grain coarsening (see Figure 9). All of the above are evidence for the ongoing microstructure relaxation.

**Figure 10:**
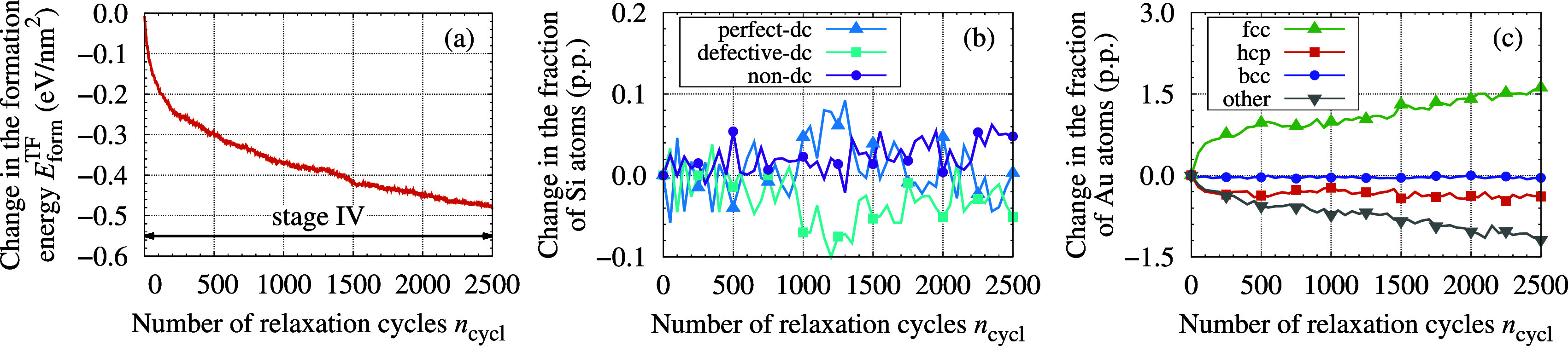
Evolution of measures characterizing the deposited TF in the final tfMC run. Note that each plot presents the change of the presented parameter(s) relative to the initial state, which was the as-deposited TF.

As seen in Figure 10, the performed final tfMC run did not allow to fully account for the microstructure changes, as both the formation energy and the structure of the TF were still changing at the end of the relaxation run. A more complete accounting for the observed changes would require performing a considerably longer—by at least an order of magnitude—tfMC run, which is computationally infeasible. We will return to this problem in the summary.

### 4.3 Characteristics of the Produced TF

We now turn to a detailed analysis of the morphology and structure of the grown TF. Here, we focus on the “relaxed” TF, i.e., the state obtained from the final tfMC run.

**Figure 11:**
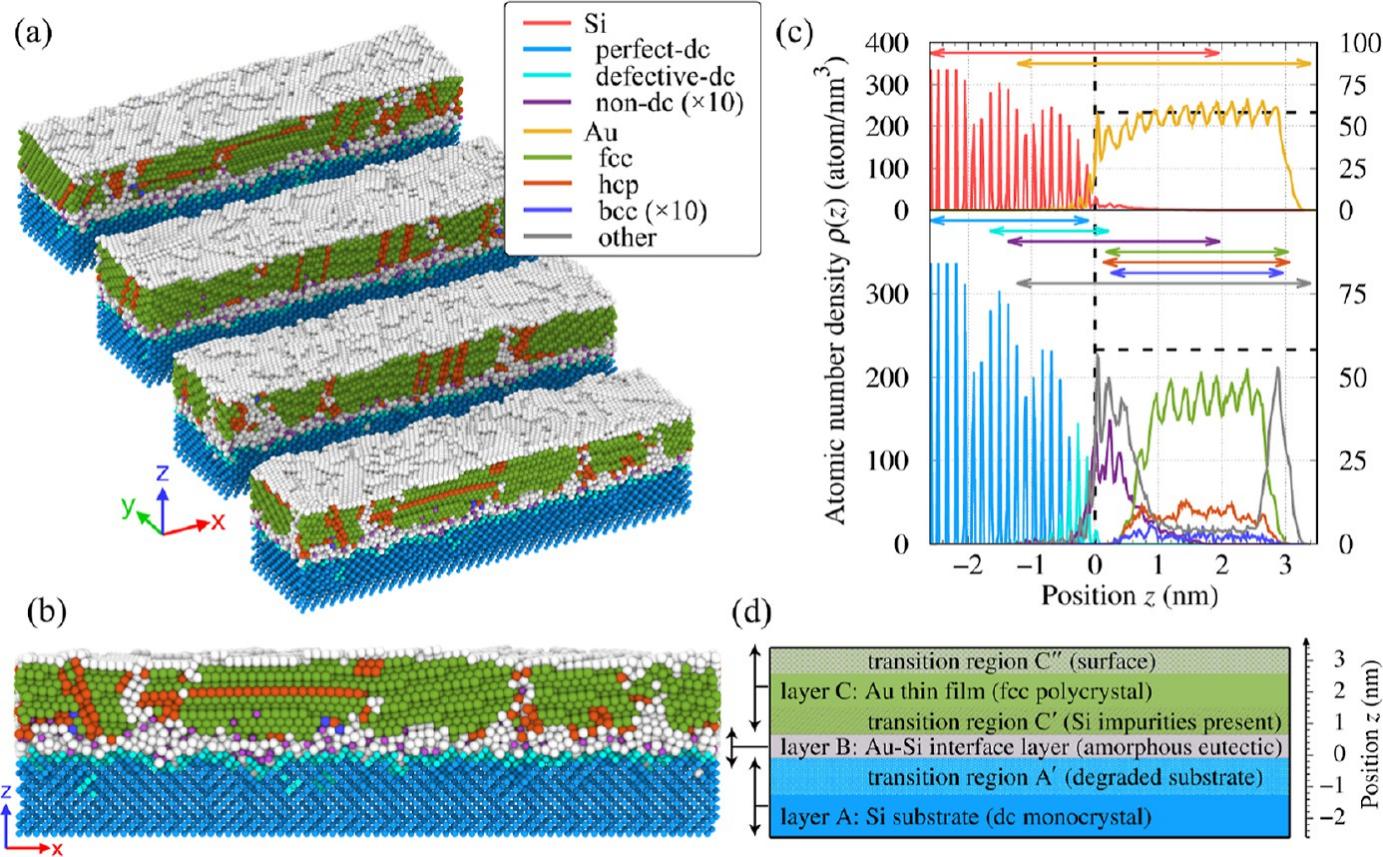
Structure and morphology of the TF obtained from the MD + tfMC simulation. Panels (a,b) visualize the system’s structure, showing several slices through the system (a) and the side view of one of the slices (b). In both panels, atoms are colored according to their local structure, with the color coding explained in the provided key. Panel (c) presents the density profiles ρ(*z*), both element-decomposed (top) and structure-decomposed. The color coding here is the same as in panels (a,b). Two vertical axes are used to read Si (left) and Au (right) data. Note that the profiles corresponding to the Si nondc and Au bcc motifs were magnified 10-fold for better readability. The vertical and horizontal dashed lines indicate the initial location of the Si substrate’s surface (*z* = 0) and the density of the bulk Au, respectively. Vertical double arrows depict *z*-ranges, where various elements/structures are observed (the corresponding ρ(*z*) is nonzero). Panel (d) is a schematic summarizing the observations made about the layered structure of the produced TF.

As illustrated in Figure 11, the TF consists of three layers with clearly different characteristics. These are the crystalline Si substrate (A), the interface Au–Si layer (B), and the polycrystalline Au layer (C). Within these main layers, three transition regions can be identified. They will be denoted as A′, C′, and C″. In what follows, we will describe in detail each of the six regions, starting from the bottom, i.e., the crystalline dc Si substrate (A).

We find that the structure of the Si substrate remained unchanged below *z* ≈ −1.7 nm, where all the Si atoms preserved their initial—perfect-dc—packing, and no other defects, like Au implants, were found.

In contrast, the top fragment of the Si substrate was considerably degraded. The presence of defects distinguishes the corresponding transition region A′ from the lower fragment of the Si substrate. They were introduced in the early deposition stage by high-energy incident Au atoms. The density profiles in Figure 11c show that these defects can be found at *z* = −1.66, −1.38, and −1.24 nm. Here, we specified the positions of the deepest-found defective-dc and nondc Si atoms, and the deepest-implanted Au atom, respectively. The first result aligns with the prediction of ref (79) (see Table 3 therein), which estimated the penetration depth of Au in silicon as 1.8 nm for the incident energy of 100 eV (same as our *E*_max_). This prediction was made based on the SRIM program, which uses a quantum mechanical treatment of ion-atom collisions.

**Table 3:** Various Simulations of TF Deposition Carried out Recently (all Employed Simulation Techniques Were Based Purely on MD) Compared to the MD + tfMC Simulation Performed in this Work, and the two Alternative Approaches (MD and MD + MD) Considered in this Work

				deposition parameters			
				deposition rate	atom flux	growth speed	
authors, reference, and year	substrate (type, dimensions and size)	thin film (type, thickness and size)	interatomic potential(s) used	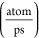	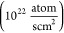	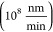	simulation method
Xie et al.^15^ 2014	dc Si (001)	Zr_*x*_Cu_1–*x*_	Tersoff,	0.5	800	120–225	MD
	2.5 × 2.5 nm^2^	4–7.5 nm	EAM, and				
	≈400 atoms	≈10,000 atoms	Lennard-Jones				
Kateb et al.^19^ 2019	fcc Cu (111)	Cu	EAM	10	1443	960–1440	MD
	7.7 × 9 nm^2^	4–6 nm	with				
	≈16,000 atoms	25,000 atoms	ZBL				
Weng et al.^20^ 2020	fcc Ni (001)	NiTi	2NN MEAM	66.7	5320	4765	MD
	11.2 × 11.2 nm^2^	3 nm					
	63,488 atoms	25,200 atoms					
Mes-adi et al.^21^ 2022	dc Si (001)	Cu	EAM	1	343	144	MD
	5.4 × 5.4 nm^2^	1.2 nm		and	and	and	
	4000 atoms	5000 atoms		10	3430	1440	
This work	dc Si (001)	Au	2NN MEAM	0.167	3.48	3.73	MD + tfMC
	21.8 × 21.8 nm^2^	3 nm		0.799	16.8	18.0	MD + MD
	64,000 atoms	80,000 atoms		2.13	44.6	47.9	MD

The high-energy collisions ejected numerous Si atoms from the top of the substrate. We found ≈2770 such atoms, corresponding to 0.86 of a single Si (001) perfect crystal plane. Consequently, the surface of the Si substrate became rough, with a roughness height in the order of 0.5 nm. Most of the released Si atoms (nearly 90%) formed the interface Au–Si layer (B), located on top of the Si substrate.

**Figure 12:**
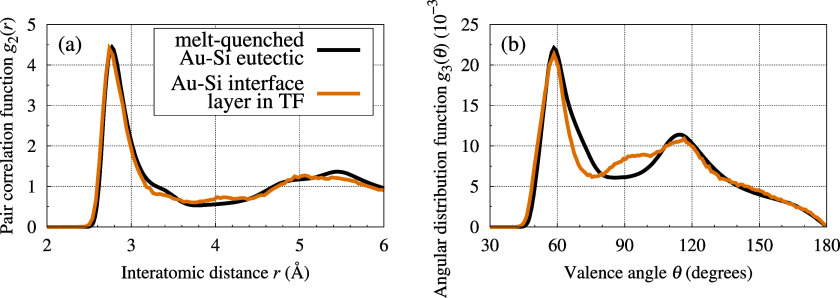
Pair correlation function *g*_2_(*r*) and angular distribution function *g*_3_(θ) of the interface Au–Si layer in the TF (yellow line) and the Au–Si eutectic melt-quenched to 300 K (black line). The calculation of *g*_3_(θ) used a 3.5 Å cutoff.

This layer has a varying height, which is typically between 0.4 and 0.6 nm, but may locally reach 1 nm. The structure of the interface Au–Si layer is predominantly disordered. The decomposed density profiles (see Figure 11c) provide the evidence. The violet and gray lines—corresponding to the Si nondc and Au other motifs, respectively—are dominating contributions within the range *z* ∈ [0.04, 0.66] nm. Another evidence is provided by Figure 12, which presents correlation functions of the Au–Si interface layer. They were calculated by accounting only for atoms residing within the range specified above. Both functions display features typical of disordered systems, like large widths and significant overlapping of the peaks, leading to nonvanishing values of the function, which leads us to conclude that the interface Au–Si layer is amorphous. By counting the number of other Au and nondc Si atoms residing within the *z* ∈ [0.04, 0.66] nm range, we estimated the composition of the interface Au–Si layer as Au_82.1_Si_17.9_, which is close to the eutectic composition (Au_80.5_Si_19.5_).

The above observations prompted us to investigate whether there are other similarities between the interface Au–Si layer and the Au–Si eutectic alloy in the amorphous state. Information on the latter’s structure was obtained from a separate MD simulation, which considered the bulk melt with an Au_82_Si_18_ composition and simulated the process of its melt-quenching from 1500 to 300 K, with a cooling rate set at 1 K/ps.

As seen in Figure 12, the correlation functions of the interface Au–Si layer closely resemble those describing the melt-quenched eutectic. There are, however, some differences. The most notable is the presence of additional features on the functions describing the Au–Si interface layer that should be attributed to the cubic ordering. These features include (i) a discernible peak in *g*_2_(*r*), centered at *r* ≈ 4.1 Å, i.e., the distance corresponding to the lattice parameter of Au, and (ii) a well-pronounced shoulder in *g*_3_(θ), positioned around θ ≈ 90°, also indicative of the cubic structures. The presence of these features can be attributed to the positioning of the Au–Si interface layer. In the TF, it is located just beneath the polycrystalline Au layer, which locally penetrates the Au–Si interface layer, influencing the existing ordering.

The above comparison allows us to assert that the interface Au–Si layer strongly resembles the melt-quenched Au–Si eutectic. This can be understood based on physical grounds, taking into account the characteristics of the deposition process, which was highly energetic. On average, a single incident atom carried an energy of *B*/2 ≈ 2 eV, which was transferred to the substrate in a localized manner during the collision. This led to a significant rise in the local temperature, exceeding 1000 K, resulting in localized melting. The localized heat was quickly dissipated into the surroundings of the deposition point, inducing the quenching effect responsible for the amorphous nature of the interface.

Above the interface Au–Si layer is the polycrystalline Au layer (C), which constitutes the bulk of the deposited TF. Depending on location, it starts between *z* = 0.22 and 0.96 nm, as the nanocrystallites formed differ in their anchoring depth, which also changes along the individual nanocrystallites. Similarly, the thickness of the nanocrystallites also varies considerably. Both effects make assigning a single thickness to the polycrystalline Au layer problematic.

In the obtained TF, the polycrystalline Au layer is formed by two large nanocrystallites (see Figure 9b), containing nearly 32,300 and 15,400 atoms. These nanocrystallites had oriented such that their (111) crystalline direction aligned with the *z*-direction. There are, however, local deviations, which may reach even 10°. As seen in Figure 11b, the stacked (111) Au planes forming the nanocrystallites are often considerably deformed (in this case, bent). This suggests that local strains and stresses are present within the polycrystalline Au layer.

**Figure 13:**
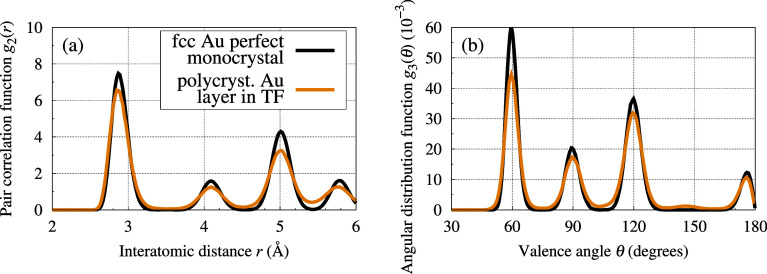
Pair correlation function *g*_2_(*r*) and angular distribution function *g*_3_(θ) of the polycrystalline Au layer in the TF (yellow line) and in the bulk Au monocrystal simulated at 300 K (black line). The calculation of *g*_3_(θ) used a 3.5 Å cutoff.

The analysis of interatomic distances and valence angles confirms this hypothesis. Figure 13 presents the *g*_2_(*r*) and *g*_3_(θ) correlation functions calculated for the polycrystalline Au layer. We also show functions obtained for the bulk Au (perfect fcc monocrystal, simulated with MD at 300 K temperature and zero pressure). Compared to this reference, the functions obtained for the polycrystalline layer in the TF display a broadening of the peaks, confirming the presence of local strains and stresses. In this work, we do not seek to quantify them as this constitutes a separate and sophisticated problem (see, e.g., ref (81)).

We found that all the nanocrystallites formed have the fcc structure. However, they contain a considerable amount of defects, such as stacking faults, twin boundaries, dislocations, and Si impurities. As seen in Figure 11c, the properties of the polycrystalline Au layer fully develop above *z* ≈ 1 nm and are nearly constant up to *z* ≈ 2.6 nm. The concentrations of defects can be quantified by counting the number of Au atoms residing within this range and displaying different packing types. Out of all Au atoms located within the range specified above, just over three-fourths (76.2%) are those with fcc packing, 15.8% constitute Au atoms with hcp packing (mostly forming the stacking faults and twin boundaries), and 7.7% are Au atoms classified as other (mostly forming grain boundaries).

**Figure 14:**
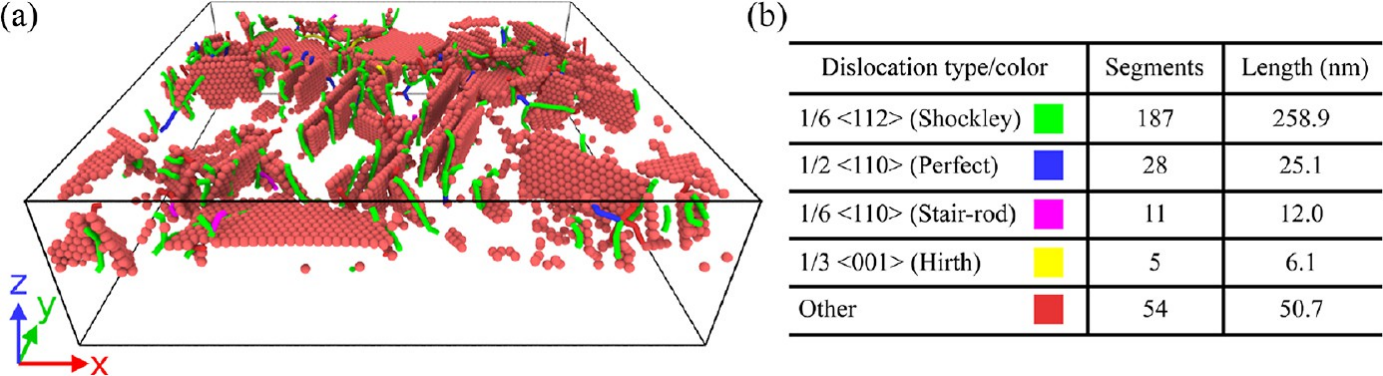
Dislocations and stacking faults. Panel (a) shows the fly view of the system, presenting a line-based representation of the dislocation network combined with a sphere-based representation of stacking faults (Au atoms with hcp local structure are shown). The table in panel (b) summarizes the results of the DXA analysis and explains the color coding used in panel (a). The number of identified dislocation segments and their total length are given for each dislocation type.

The polycrystalline Au layer contains many dislocations (see Figure 14). From DXA, we found nearly 300 dislocation segments, with the total length of dislocation lines being ≈350 nm. The 1/6⟨112⟩ Shockley partial dislocations dominate (we found nearly 190 dislocation segments, with ≈270 nm total length), bounding numerous stacking faults and twin boundaries (nearly 80). The obtained TF is, therefore, characterized by a very high dislocation density, which in the considered case reached 2.5 × 10^19^ m^–2^. This value is 3–4 orders higher than dislocation densities typical for strongly deformed bulk materials. However, it is comparable to dislocation densities measured in gold nanocontacts evaporated on silicon: 2.3 × 10^19^ m^–2^ was reported in ref (82).

Out of all Si atoms ejected from the substrate, a considerable fraction (nearly 300 atoms) had diffused into the polycrystalline Au layer. This allows defining—within layer C—a transition region C′. Its distinguishing feature is the presence of a noticeable amount of nondc Si atoms. As seen from the density profile (see the violet line in Figure 11c), these atoms are observed up to *z* ≈ 2 nm. It is worth mentioning that the Si atoms that diffused into the polycrystalline Au layer prefer to form chemically ordered motifs. Nearly half (≈150 atoms) displayed L1_2_ ordering, a motif typical for the Au_3_Si alloy, a metastable compound.^83−85^

As seen in Figure 11c, the number of Au atoms classified as other increases sharply above *z* = 2.6 nm. This effect can be attributed to the presence of the surface, which can be regarded as another transition region (C″). Many steps are visible on the surface, but they have irregular character (see Figure 8b). The surface roughness can be described by specifying the difference between the maximum and minimum *z* coordinates of atoms forming the surface, which in the considered case was 0.8 nm. Because of this effect, the thickness of the deposited TF also varies considerably, changing between 2.6 and 3.4 nm, with an average of roughly 3 nm.

## 5 Characteristics of the Proposed Approach

### 5.1 Accessible Time Scales

In ref (31) Bal and Neyts addressed the problem of time scale associated with the tfMC simulation. By performing numerical experiments for a variety of systems subjected to various conditions, they demonstrated that the effective time step ⟨Δ*t*^tfMC^⟩ is proportional to  and to 1/*T*, and not to Δ*r*_max_^tfMC^ and  as would be predicted by eq 1; therefore, questioning the validity of this formula, derived by Mees et al. in ref (30).

With the above in mind, to reliably quantify the benefits of tfMC, we determined ⟨Δ*t*^tfMC^⟩ from scratch for the considered system, i.e., the Au TF. Our determination also accounted for the fact that the ⟨Δ*t*^tfMC^⟩ parameter may vary in the simulated process, with different values at different deposition stages.

We determined ⟨Δ*t*^tfMC^⟩ through simulations of long-time evolution. In these simulations, configurations of TF obtained from MD + tfMC were subjected to MD and tfMC runs, in which their further evolution and the corresponding relaxation were simulated. These simulations consisted of 10^7^ steps each. All the MD runs used the same time step of Δ*t*^MD^ = 1 fs. All the tfMC runs used the same maximum step length of Δ*r*_max_^tfMC^ = 0.1 Å.

**Figure 15:**
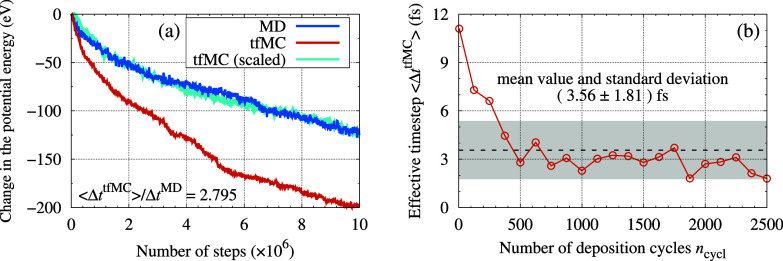
Estimation of the effective time step ⟨Δ*t*^tfMC^⟩. Panel (a) presents the evolution of the potential energy in MD and tfMC runs performed for the structure of TF obtained after 1500 cycles. The light blue curve shows how the potential energy in the tfMC run would compare with the MD run if it was assumed that a single tfMC step corresponds to multiple, namely ⟨Δ*t*^tfMC^⟩/Δ*t*^MD^≈2.8, MD steps. Panel (b) presents how the effective time step ⟨Δ*t*^tfMC^⟩ varied during the deposition, showing it as a function of *n*_cycl_. The dashed black line shows the mean value of the determined ⟨Δ*t*^tfMC^⟩, while the shaded area represents one standard deviation from the mean value.

The method used for finding ⟨Δ*t*^tfMC^⟩ is illustrated in Figure 15a, which compares the evolution of the system’s potential energy in the MD and tfMC simulations starting from the same structure, i.e., the TF obtained after 1500 deposition cycles. In the MD run, after 10^7^ steps, the energy decreased by roughly 125 eV. In the tfMC run, a similar energy decrease was observed after 3.6 × 10^6^ steps. Knowing that Δ*t*^MD^ = 1 fs, we estimate the effective time step of tfMC as ⟨Δ*t*^tfMC^⟩ = 2.8 fs.

Similar analysis was performed for structures of TF corresponding to other stages of deposition, i.e., after 5, 125, 250..., 2500 cycles. This allowed finding how the ⟨Δ*t*^tfMC^⟩ parameter depends on *n*_cycl_. The obtained results are summarized in Figure 15b. tfMC was the most effective at the beginning of the deposition (i.e., up to 250 cycles), where the measured ⟨Δ*t*^tfMC^⟩ reached 7–11 fs. Subsequently, the measured ⟨Δ*t*^tfMC^⟩ decreased considerably, to between 2 and 4 fs until the very end of deposition. Because this considerable decrease in the tfMC’s efficiency is undesired, it merits explanation.

The presented estimates of ⟨Δ*t*^tfMC^⟩ are based on a global measure (potential energy). Therefore, the determined ⟨Δ*t*^tfMC^⟩ reflects the ability of the tfMC to speed up the observed relaxation in an average (system-wide) sense. As such, the measured ⟨Δ*t*^tfMC^⟩ is sensitive to effects such as a change in the size of the domain where the operation of the tfMC algorithm was efficient.

**Figure 16:**
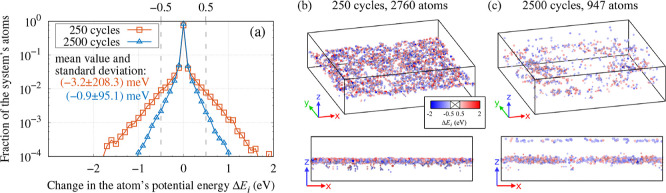
Domain of the tfMC’s efficient operation. Results of atom-resolved analysis are presented, which considered the changes in the potential energies of atoms Δ*E*_*i*_ = *E*_*i*_^final^ – *E*_*i*_^init^, observed in the long-time tfMC runs. Panel (a) compares distributions of Δ*E*_*i*_ obtained in tfMC runs, which started from structures of TF obtained after 250 and 2500 cycles. Values given within the plot specify the mean value and standard deviation of Δ*E*_*i*_. Panels (b,c) present the corresponding TFs after the long-time tfMC runs, showing only the atoms whose potential energies changed considerably, i.e., by |Δ*E*_*i*_| ≥ 0.5 eV. The number of such atoms is given in the figure. Atoms were colored according to Δ*E*_*i*_ (see the provided color key).

To demonstrate that this is indeed what happened, in Figure 16, we present the results of a detailed—atom-resolved—analysis. Panel (a) shows how the potential energies of individual atoms changed during the long-time tfMC runs performed for structures of TF obtained after *n*_cycl_ = 250 and 2500 cycles. The distribution corresponding to *n*_cycl_ = 250 is much broader, meaning the tfMC algorithm operated on a larger fragment of the system in the early deposition stage. This is seen in Figure 16b, which shows that the tfMC algorithm operated on the entire deposited TF at this stage.

Subsequently, once a TF of considerable thickness was deposited and a polycrystalline Au layer was formed, the domain of tfMC’s efficient operation spanned the surface of the TF and the boundary between the polycrystalline Au and the interface Au–Si layers (see Figure 16c), i.e., only those regions where further relaxations were still possible. The remaining fragments of the system had been relaxed in earlier cycles. This reduction in the domain of efficient operation explains the decreasing trend in the ⟨Δ*t*^tfMC^⟩ vs *n*_cycl_ dependence. However, there is also another reason.

Reference (31) reported that the efficiency of tfMC is system- and process-dependent. For instance, it has been observed that tfMC performs better (yielding longer ⟨Δ*t*^tfMC^⟩) when simulating surface phenomena, such as atom hopping in surface diffusion. Furthermore, it has been noted that it is less effective when investigating processes occurring in the bulk, performing particularly poorly for disordered systems.^32^ These observations allow attributing the noted efficiency decrease to the fact that both the nature of the simulated process and the characteristics of the simulated system changed significantly throughout the deposition. Here, we emphasize the importance of findings from Section 4, especially those pertaining to the character of the deposition process (different in stages I, II, and III) and the structure of the produced TF (different within layers A, B, and C).

The above analysis shows that tfMC considerably extends the time scale accessed in the simulation. The determined ⟨Δ*t*^tfMC^⟩ vs *n*_cycl_ dependence allows estimating the total physical time of the performed MD + tfMC deposition simulation as 0.483 μs, with tfMC accounting for 0.446 μs. Knowing the total physical time lets us calculate the growth speed, which was 3.7 × 10^8^ nm/min (on average). This value is still extremely large compared to experiments, where growth speeds are of the order of nm/min,^86,87^ with typical atom fluxes being of the order 10^15^ atom/s/cm^2^. However, in the performed hybrid MD + tfMC simulation, the growth speed and atom flux were two to 3 orders of magnitude smaller than those used in simulations carried out to date by other authors. To support this statement, in Table 3 we compare the MD + tfMC simulation performed in this work with other recent works that simulated the deposition of various TFs, employing simulation protocols that were based purely on MD.

### 5.2 Comparison with other Simulation Methods

In the preceding subsections we demonstrated that the proposed hybrid MD + tfMC method leads to a picture of TF growth which accounts for effects (like the formation of a nanostructure) that require long times to occur. We attribute this success to tfMC, particularly its ability to extend the time scale reached in a simulation and the improved probing of the configurational space that it offers. To demonstrate that the use of tfMC is critical, we will now compare the results obtained with the hybrid MD + tfMC method against two alternative approaches: MD and MD + MD. Both were described in Section 3.1.

In these two approaches, the total physical time of the entire deposition was 0.038 μs (MD) and 0.10 μs (MD + MD), which is nearly 13- and 5-times shorter, respectively, than in the MD + tfMC simulation (0.48 μs). The computational efforts of the MD + MD and MD + tfMC simulations were comparable, while that of the MD simulation was approximately two times smaller.

**Figure 17:**
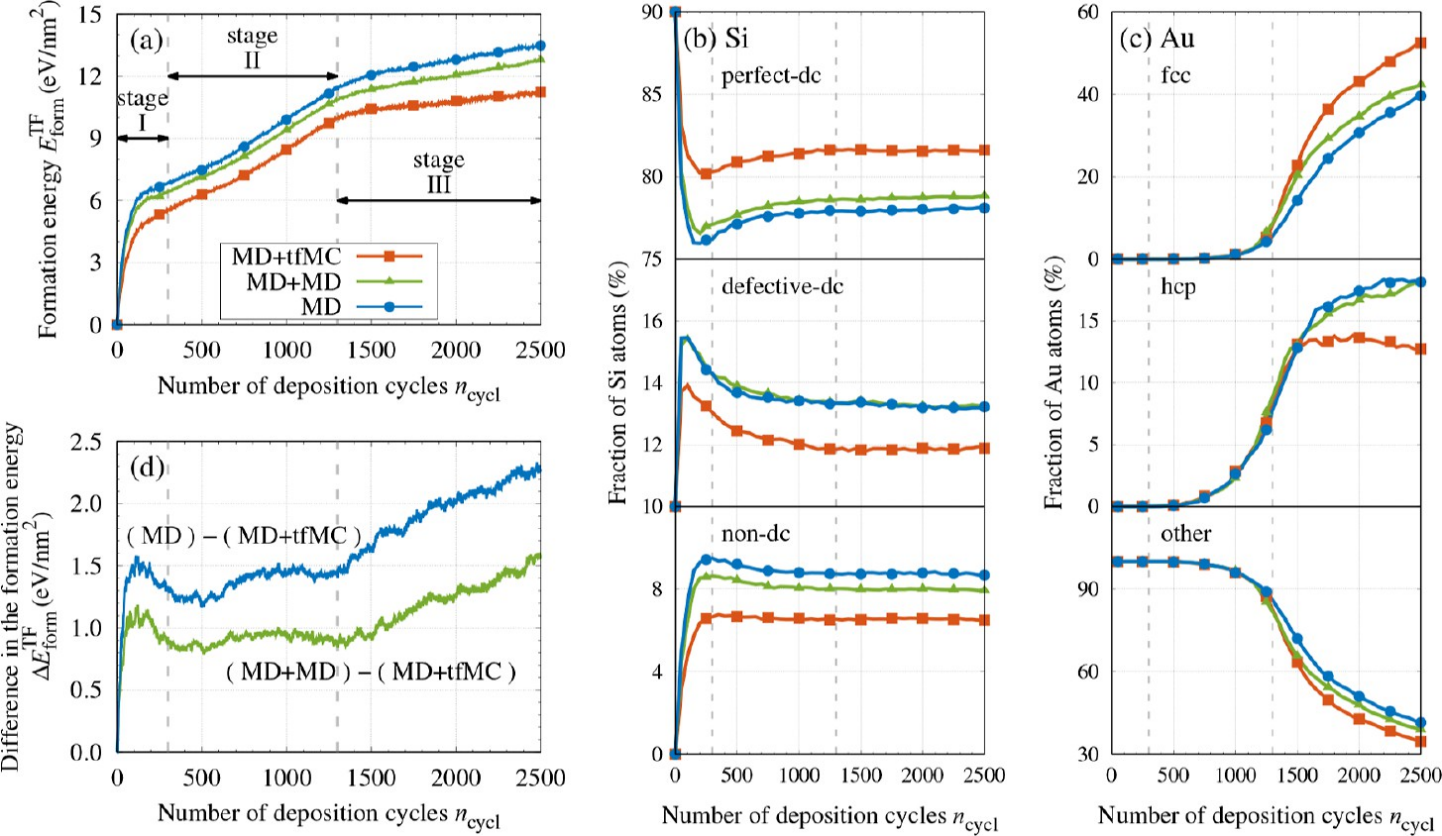
Influence of the simulation method on the energy [panels (a,d)] and structure [panels (b,c)] of the growing TF. Panels (a–c) are analogous to panels (a–c) from Figure 4. The data for the MD + tfMC method is repeated, and results from the alternative approaches (MD and MD + MD) are added to the plots for comparison. The key in panel (a) also applies to panels (b,c). Panel (d) presents the differences in formation energies (as described within the plot).

Figure 17a presents how the formation energy of the TF evolved. In all three cases, the *E*_form_^TF^ vs *n*_cycl_ dependence has a similar character, with the slope changing between stages I–III, identified and described earlier. This similarity shows that all three approaches probed the same process. This conclusion is further supported by the structural analysis, which showed that, in all three approaches, the structure of the TF also evolved similarly, as evidenced by the similarity of all six triples of characteristics shown in Figure 17b,c. The observed differences are only quantitative in character, and the three TFs produced differ mainly in the fraction of atoms displaying the energetically preferred packing types—fcc for Au and perfect-dc for Si—which were the highest in the TF obtained from the MD + tfMC simulation. Consequently, the TFs obtained from the MD + MD and MD simulations contained more atoms belonging to the remaining classes (see Table 4 for values).

**Table 4:** Measures Characterizing the TFs Obtained with the Three Considered Approaches^a^

	simulation method		
	MD + tfMC	MD + MD	MD
formation energy (eV/nm^2^)	11.23	12.76	13.52
local structures in the entire system (%)			
Si atoms			
perfect-dc	81.61	78.85	78.10
defective-dc	11.89	13.22	13.22
nondc	6.50	7.93	8.68
Au atoms			
fcc	52.40	42.29	39.59
hcp	12.74	18.10	18.17
bcc	0.37	0.71	0.77
other	34.49	38.90	41.47
local structures within the polycrystalline Au layer (%)			
Au atoms			
fcc	75.13	61.43	57.70
hcp	16.16	23.99	24.36
bcc	0.43	0.90	1.01
other	8.28	13.68	16.93

a In all three cases, the presented results describe the TF’s state immediately after the deposition is completed. Therefore, the data for the MD + tfMC method is a repeat of the data from Table 1 (the “as-deposited” column). The same conventions as in Table 1 are used, with all structural analysis results expressed in percentages. Here, in addition to the structural analysis carried out globally (throughout the entire system), we also report on the structural analysis carried out within the polycrystalline Au layer (see text).

The differences visible in the structure also manifest themselves in the energy. The TF obtained from the MD + tfMC simulation has *E*_form_^TF^ = 11.2 eV/nm^2^. This value is 1.5 and 2.3 eV/nm^2^ lower, respectively, than *E*_form_^TF^ obtained from the MD + MD and MD simulations. Pairwise comparison of *E*_form_^TF^ vs *n*_cycl_ dependences (see Figure 17d) reveals that this difference mostly appeared in the initial stage of the growth (up to 150 deposition cycles), after which the *E*_form_^TF^ of TFs obtained from the MD + MD and MD simulations were already by 1.1 and 1.5 eV/nm^2^ higher. This shows the importance of using tfMC at the beginning of the deposition, where—as concluded in Section 5.1—it was the most effective, providing—as compared to MD and MD + MD—a less degraded surface of the Si substrate. To better illustrate the observed differences, we note that in the MD + tfMC simulation, the fraction of Si atoms whose structure changed from perfect-dc was 8.4%. In the MD + MD and MD simulations, this fraction reached 11.2% and 11.9%, respectively, meaning an extra 0.56–0.70 of the Si (001) perfect plane was destroyed. In contrast, all three approaches provided a similar picture of Au atoms’ implantations, with the deepest implant found at *z* = −1.22 nm.

The tfMC method also greatly influenced the polycrystalline Au layer. As seen in Figure 17a, in stage III (i.e., above *n*_cycl_ = 1300), in the MD + tfMC simulation, *E*_form_^TF^ increased slower than in the MD + MD and MD simulations. The corresponding slopes were 0.90, 1.41, and 1.42 (all in meV/nm^2^/cycle). This suggests that the MD + tfMC simulation provided a more ordered polycrystalline Au layer.

**Figure 18:**
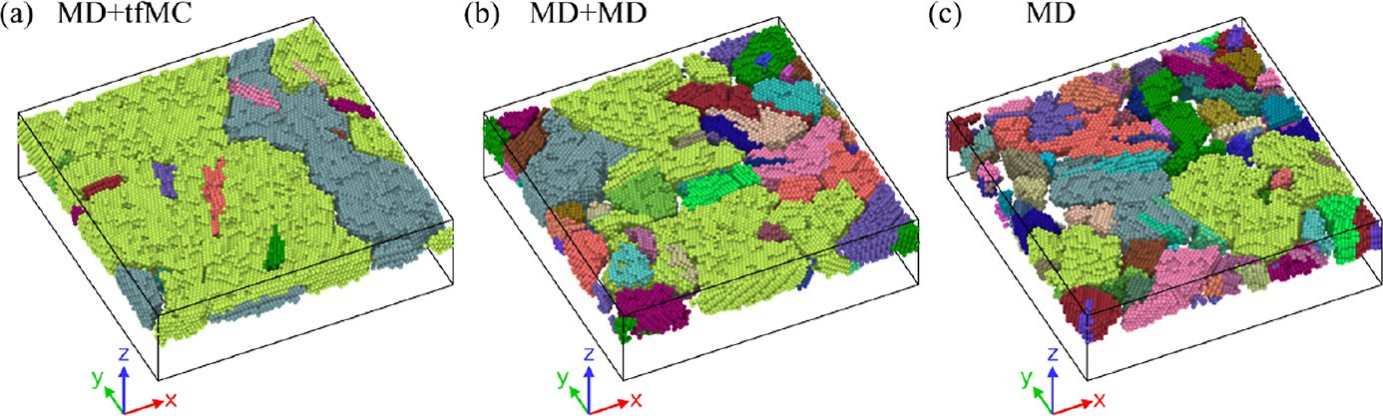
Grain structure of TFs obtained with the three considered approaches. The “as-deposited” TFs are visualized in the same way as in Figure 9. The identification of grains was also carried out using the same method.

This hypothesis was confirmed by analyzing the decomposed density profiles (analysis analogous to that presented in Section 4.3, not shown for brevity). It revealed that within the polycrystalline Au layer, the differences between the three obtained TFs were even more prominent than those visible in Figure 17c. We found that in the TF obtained from the MD + tfMC simulation, within the polycrystalline Au layer (i.e., between *z* = 1 and 2.6 nm), 75.1% of Au atoms displayed fcc packing. This value is significantly higher than those obtained with MD + MD (61.4%) and MD (57.7%). Consequently, in the TF obtained from the MD + tfMC simulations, the polycrystalline Au layer contained a lower fraction of Au atoms with hcp packing (16.1%) and Au atoms classified as other (8.3%). In the TFs obtained from the MD + MD and MD simulations, these fractions were higher by ≈8 p.p. (hcp Au atoms, in both methods) and by 5.4–8.6 p.p. (other Au atoms, MD + MD and MD methods, respectively). These observations indicate that the TFs obtained with the alternative approaches contained more stacking faults and other structural defects, like grain boundaries. This is well visible in Figure 18, which visualizes the grain structures of all TFs, showing that the MD + tfMC simulation provided a better-developed microstructure, with the polycrystalline Au layer being mainly formed from two large nanocrystallites (and 13 nanocrystallites in total). In the TFs obtained from the alternative approaches, we found as many as 36 (MD + MD) or 61 (MD) nanocrystallites.

**Figure 19:**
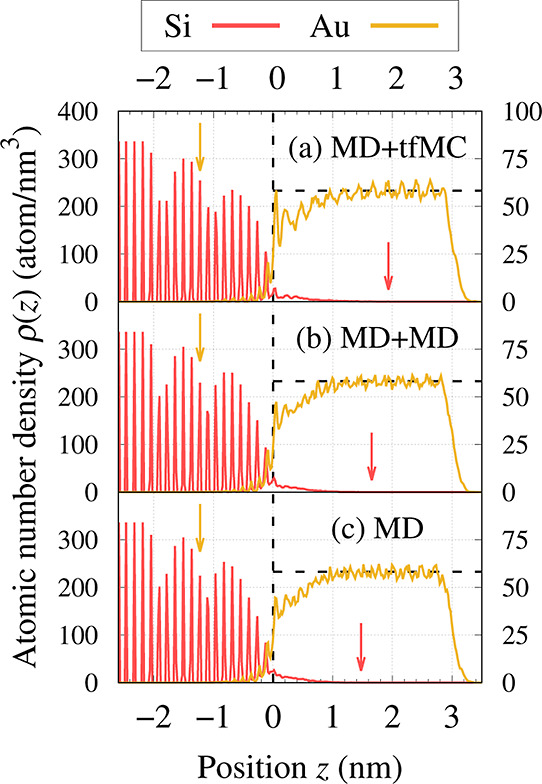
Density profiles of TFs obtained with the three considered approaches. The presentation method is the same as in Figure 6. Density profiles of the “as-deposited” TFs are presented.

Regarding the interface Au–Si layer, all three approaches yielded a composition similar to eutectic Al_80.5_Si_19.5_, namely Au_82.3_Si_17.7_ (MD + tfMC), Au_81.3_Si_18.7_ (MD + MD), and Au_80.4_Si_19.6_ (MD). As clearly visible, in the TFs obtained from the MD + MD and MD approaches, the content of Si was slightly (by ≈1–2 p.p.) higher within the interface Au–Si layer than in the MD + tfMC simulation. This effect can be explained by referring to our earlier observation that in the MD + MD and MD simulations, the top layer of the Si substrate was degraded to a greater extent. Therefore, more Si atoms were ejected from it. Subsequently, these excess Si atoms mainly accumulated in the Au–Si interface layer, as their diffusion into the polycrystalline Au layer was limited by the shorter time scale of MD + MD and MD simulations. Consequently, the Si diffusion depth was also visibly lower in the MD + MD and MD simulations. As seen in Figure 19, the Si atom which diffused the most into the polycrystalline Au layer, was found at *z* = 1.66 nm (MD + MD) and 1.48 nm (MD), and these values are by 0.28–0.46 nm lower than in the MD + tfMC simulation (*z* = 1.94 nm).

Despite significant differences in the energetics and structure, we found that the three TFs obtained did not differ considerably in density. The density profiles ρ(*z*) presented in Figure 19 show that within the polycrystalline Au layer, the density of all three TFs was similar and close to the density of the bulk Au (depicted with a black dashed line). However, the Au density profile obtained from the MD + tfMC simulation has more pronounced peaks above *z* = 0. As these peaks correspond to the stacked (111) Au planes, the observed feature also reveals a more ordered character of the TF obtained from the MD + tfMC simulation.

Among other noted differences, we found that in the MD + tfMC simulation, the sticking coefficient was visibly higher. As stated earlier, in this simulation, only 0.65% of the incident Au atoms failed to deposit. This percentage reached 0.81% and 0.84% in the MD + MD and MD simulations, respectively. We attribute this effect to the fact that in the MD + MD and MD simulations, the surface of the growing TF was at all times relaxed to a lower extent and—therefore—was more susceptible to sputtering.

## 6 Conclusions

### 6.1 Summary

In this work, we proposed a novel approach to simulating the deposition of thin films from the gas phase, which combines two simulation techniques, namely molecular dynamics (MD) and time-stamped force-bias Monte Carlo^30^ (tfMC). In the employed simulation protocol, the two methods are used alternately, with the entire simulation being a series of cycles. Each cycle consists of one MD and one tfMC run, and models the deposition of a small portion of atoms and relaxations that take place immediately after. MD—which provides fine (femto- and subfemtosecond) temporal resolution—is used to resolve fast events, i.e., the collisions between the atoms being deposited and the substrate. The subsequently applied tfMC models slow relaxation processes which occur between collisions, on time scales considerably longer than those accessible to and covered by MD. The proposed approach also accounts for other aspects essential for realistic deposition modeling. For example, the incident atoms are described with realistic energy and angle distributions.

To demonstrate the practicability of the proposed hybrid MD + tfMC approach, we employed it to study the physical vapor deposition of a thin (3 nm thick) Au film on crystalline Si, carried out in a vacuum under isothermal conditions at 300 K. A comparison with two alternative approaches, both purely MD, showed that using tfMC is very beneficial. The MD + tfMC approach considerably (by a factor of 5) extended the accessed time scale, yielding a thin film with a more ordered structure and a better-developed microstructure. Numerical experiments allowed estimating the time scale of the performed MD + tfMC simulation as 0.48 μs. Therefore, the deposition rate could be made two to 3 orders of magnitude smaller than that of other deposition simulations carried out in the last years.

We also described the growth of Au on crystalline Si, revealing that it consists of four distinct stages (I–IV). In stage I, considerable degradation of the Si substrate’s surface occurred, leading to the creation of a mixed interface layer containing Au and Si atoms. This layer grew in stage II, by the end of which stable nuclei of the Au fcc phase were formed. In stage III, an ordered growth of the formed Au nanocrystallites was observed and it proceeded in a fashion close to the Frank-van der Merwe (layer-by-layer) mode. After the deposition was finished, i.e., in stage IV, the produced TF relaxed through various microstructure reorganization mechanisms.

The thin film obtained from the simulation was analyzed from a number of perspectives. It was found that its structure varied across its six layers, namely the three main layers (A, B, C) and three transition regions (A′, C′, C″), stacked in the sequence A–A′–B–C′–C–C′′. The Si substrate (A) was considerably degraded to a depth locally reaching 1.7 nm. Above the corresponding transition region (A′), an interface Au–Si layer (B) was created, with nearly eutectic composition (Au_82_Si_18_), varying height (0.4–1 nm), and amorphous structure. Upon this layer, a polycrystalline fcc Au layer grew, with a height varying between 2 and 2.5 nm. Its bottom fragment (C′) contained a considerable number of Si atoms, which diffused into the polycrystalline Au layer to a depth locally reaching 1.5 nm. The polycrystalline Au layer (C) had an fcc structure with multiple defects, such as stacking faults, dislocations, and grain boundaries. The Au nanocrystallites were oriented such that their (111) crystalline direction aligned with the surface normal. The surface of the polycrystalline Au layer (C″) was characterized by considerable roughness, with a height reaching 0.8 nm.

### 6.2 Discussion and Future Work

The carried out MD + tfMC simulation covers time scales spanning 10 orders of magnitude, ranging from 10^–17^ s (the time resolution with which the collisions of the most energetic incident atoms were time-integrated) to 10^–7^ s (the length of the entire simulation, consisting of the actual deposition and postdeposition relaxation, reached 0.7 μs). Reaching a μs scale allowed observing the onset of microstructure reorganization. The final relaxation run did not allow simulating this process completely, as this would require a considerably longer run, which remains computationally infeasible with today’s resources.

With the above in mind, it would be beneficial to extend the time scale of the simulation further. As a potential solution, we identify the application of simulation boosting techniques designed to cover the above-μs time scale. One promising technique is the recently proposed collective variable-driven hyperdynamics^51^ (CVHD). Recent works have shown that CVHD allows simulations to reach time scales from milliseconds^88^ to seconds.^89,90^ We have already begun work on extending the simulation protocol presented in this study to incorporate CVHD in addition to MD and tfMC. We anticipate that employing such an approach will significantly extend the simulation’s time scale, allowing us to further decrease the deposition rate and to enable a more comprehensive account of the observed microstructure reorganization.

This work once again demonstrated the usefulness of atomistic simulations. The performed simulations and analyses provided new insights into mechanisms governing the deposition and growth of TFs. Simulation yields a picture with very fine spatial and temporal resolution, allowing the observation of phenomena, such as local structural transformations, occurring on length- and time scales that remain unattainable in experimental observations. Here, we note that even dedicated spectroscopic techniques face significant difficulties when characterizing very thin films (see, e.g., refs (2 and 91)). The lack of experimental data is the reason why we did not compare the obtained results against experiments. However, we expect that such a comparison—made indirectly through the comparison of thermodynamic properties—will become possible in the near future.

The reason we chose Au TF as an example system is the experimental study carried out by Łapiński et al.^86,91^ They observed that Au thin films obtained from PVD transform upon thermal treatment, disintegrating into a discontinuous film composed of so-called nanoislands. References (86 and 91) reported that this transformation occurs at temperatures considerably lower than would otherwise be expected, even when accounting for the size dependence of the melting point. The origins of this behavior were unclear and difficult to identify solely from experiment. From this perspective, this work, which focuses on obtaining a realistic structure of TF in silico, constitutes a first step in planned broader research. Its main scope is to support the cited experimental works. We anticipate that the modeling studies initiated in this work will align with experimental findings, offering an atomistic-scale explanation for the observed low-temperature reorganization. The associated problems are the focus of our ongoing research.

## Supporting Information Available

Study of the effect of the maximum displacement parameter Δ*r*_max_^tfMC^ on the results of the MD + tfMC simulation (PDF)
