# Supplementary material for: Novel Method for Realistically Simulating the Deposition of Thin Films from the Gas Phase and its Application to Study the Growth of Thin Gold Film on Crystalline Silicon
Source: J Chem Theory Comput. 2025 Apr 30;21(9):4792–814. doi: 10.1021/acs.jctc.5c00319 (PMC12079803; doi:10.1021/acs.jctc.5c00319)
Supplement: Supplementary file 1 — ct5c00319_si_001.pdf [file ct5c00319_si_001.pdf]

Supporting Information: A novel method for realistically simulating the deposition of thin films from the gas phase and its application to study the growth of thin gold film on crystalline silicon

Szymon Winczewski,[*,†] Jacek Dziedzic,[†,‡] Marcin Łapiński,[†] and Jarosław Rybicki[†,¶]

†*Faculty of Applied Physics and Mathematics, Gdansk University of Technology, Narutowicza 11/12, 80-233 Gdańsk, Poland*

‡*School of Chemistry, University of Southampton, Highfield, Southampton SO17 1BJ, United Kingdom*

¶*TASK Computer Centre, Gdansk University of Technology, Narutowicza 11/12, 80-233 Gdańsk, Poland*

E-mail: szymon.winczewski@pg.edu.pl

S1 Influence of the maximum displacement parameter

We now analyze how the $\Delta r_{\mathrm{max}}^{\mathrm{tfMC}}$ parameter influences the picture obtained from the simulation. Figure S1 compares the evolution of the formation energy $E_{\mathrm{form}}^{\mathrm{TF}}$ in the MD+tfMC simulations carried out at various values of $\Delta r_{\mathrm{max}}^{\mathrm{tfMC}}$. The curve obtained from the MD method is also presented, as it formally can be regarded as the $\Delta r_{\mathrm{max}}^{\mathrm{tfMC}} = 0$ case. All six $E_{\mathrm{form}}^{\mathrm{TF}}$ vs. n_{cycl} dependences display qualitatively similar character, with a monotonically increasing behavior and slope changing between the three stages of growth (I-III). This similarity shows that all simulations probed a similar process. However, some quantitative differences are observed, particularly for the two highest values of $\Delta r_{\mathrm{max}}^{\mathrm{tfMC}}$, *i.e.*, 0.20 and 0.25 Å.

As seen in Figure S1, increasing $\Delta r_{\mathrm{max}}^{\mathrm{tfMC}}$ from 0 to 0.15 Å lowered the formation energy in all three stages. However, using a higher $\Delta r_{\mathrm{max}}^{\mathrm{tfMC}} = 0.20$ Å led to a TF film whose final formation energy was higher than for $\Delta r_{\mathrm{max}}^{\mathrm{tfMC}} = 0.15$ Å (see Table S1 for numerical values). The dependence obtained for 0.20 Å crossed the curve obtained for 0.15 Å between $n_{\mathrm{cycl}} \approx$ 2200-2300 cycles. For the highest considered value $\Delta r_{\mathrm{max}}^{\mathrm{tfMC}} = 0.25$ Å, this crossing occurred even earlier, at ≈1500 cycles, *i.e.*, at the onset of stage III. Within this stage, the $E_{\mathrm{form}}^{\mathrm{TF}}$ vs. n_{cycl} dependence obtained for $\Delta r_{\mathrm{max}}^{\mathrm{tfMC}} = 0.25$ Å visibly deviates from the trend observed for other tested $\Delta r_{\mathrm{max}}^{\mathrm{tfMC}}$ values, clearly tending toward higher $E_{\mathrm{form}}^{\mathrm{TF}}$.

The above observations can be explained by referring to the structural analysis (see Table S1). Increasing $\Delta r_{\mathrm{max}}^{\mathrm{tfMC}}$ from 0 to 0.15 Å resulted in the production of a more ordered TF, containing a higher fraction of fcc Au atoms. As opposed to that, the application of $\Delta r_{\mathrm{max}}^{\mathrm{tfMC}} = 0.20$ and 0.25 Å provided TFs with visibly lower content of fcc Au atoms. In addition, these TFs also contained a higher amount of non-dc Si atoms, displaying a higher concentration of these atoms within the polycrystalline Au layer, as revealed from the analysis of the density profiles $\rho(z)$ (not shown for brevity). At the same time, the TFs obtained for $\Delta r_{\mathrm{max}}^{\mathrm{tfMC}} = 0.20$ and 0.25 Å were characterized by a less-developed grain structure, containing a considerably higher number of smaller grains than TFs obtained for

0.15 and 0.10 Å (see Figure S2, compare with Figure 18a in the main text).

The effects observed for $\Delta r_{\mathrm{max}}^{\mathrm{tfMC}} = 0.20$ and 0.25 Å should be considered as artifacts of the simulation method used resulting from an incorrect choice of the $\Delta r_{\mathrm{max}}^{\mathrm{tfMC}}$ parameter, which – by being too high – lowered the barriers for Si diffusion too significantly, distorting the relative importance of this process, and allowing the Si atoms to diffuse too freely into the Au TF being deposited. The excess Si atoms delayed the crystallization of the Au polycrystalline layer. This effect is visible in Figure S1, especially in the curve for $\Delta r_{\mathrm{max}}^{\mathrm{tfMC}} =$ 0.25 Å, whose final slope settled visibly later. By acting as defects, the excess Si atoms also limited the possible ordering of the polycrystalline Au layer, preventing the formation of larger nanocrystallites (see Figure S2c).

The results of this section support our choice of $\Delta r_{\mathrm{max}}^{\mathrm{tfMC}} = 0.10$ Å. However, 0.15 Å could also be taken, or even a slightly higher value, provided that it was lower than 0.20 Å. Our finding supports the suggestion formulated in Ref. S1, which stated that $\Delta r_{\mathrm{max}}^{\mathrm{tfMC}}$ should not exceed 5-10% of the shortest interatomic distance. The bound we found (0.20 Å) constitutes 8.4% of the shortest Si-Si distance (2.361 Å).

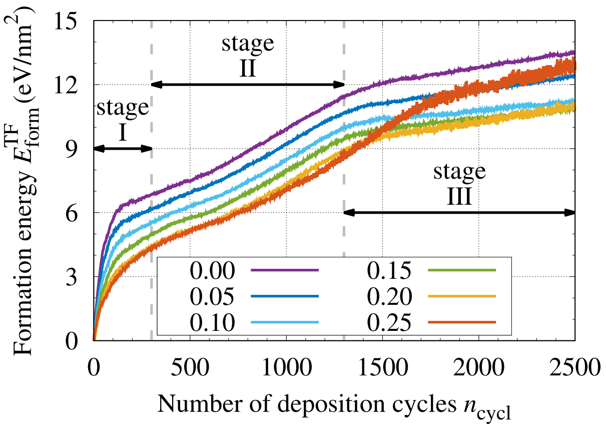

Figure S1: Influence of the maximum displacement parameter $\Delta r_{\mathrm{max}}^{\mathrm{tfMC}}$ on the evolution of the formation energy $E_{\mathrm{form}}^{\mathrm{TF}}$ during the deposition.

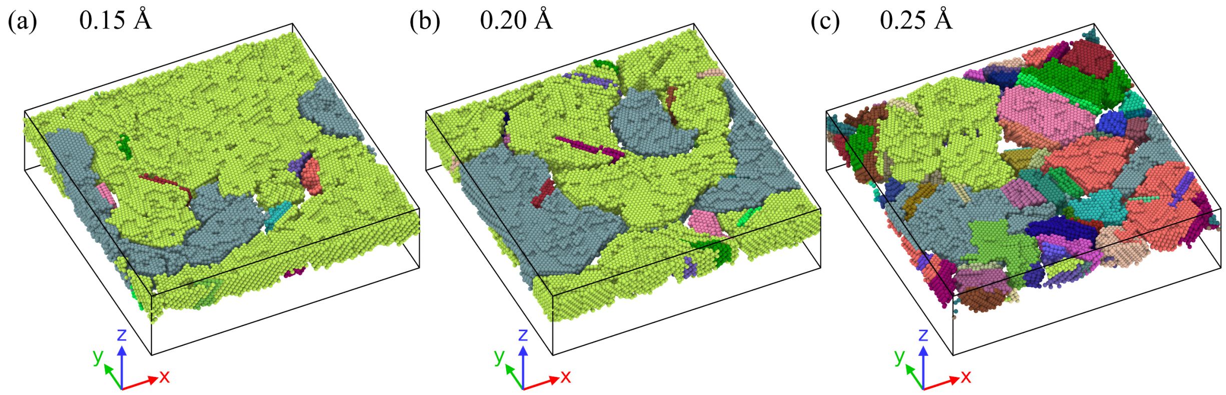

Figure S2: Influence of the maximum displacement parameter $\Delta r_{\mathrm{max}}^{\mathrm{tfMC}}$ on the grain structure of TF obtained from the MD+tfMC simulation. The "as-deposited" TFs are visualized in the same way as in Figure 9 in the main text.

Table S1: Effect of the maximum displacement parameter $\Delta r_{\mathrm{max}}^{\mathrm{tfMC}}$ on the characteristics of TF obtained from simulation. The presented results describe the TF's state immediately after the deposition is completed (the "as-deposited" TF). The data for the MD method and MD+tfMC method with $\Delta r_{\mathrm{max}}^{\mathrm{tfMC}} = 0.10$ Å are repeats of the data from Table 4 in the main text. Here, the same convention is used, with all structural analysis results expressed in percentages.

Method	MD	MD+tfMC				
$\Delta r_{\mathrm{max}}^{\mathrm{tfMC}}$ (Å)	0.00	0.05	0.10	0.15	0.20	0.25
formation energy (eV/nm^2)	13.52	12.38	11.23	10.86	10.98	12.93
local structures in the entire system (%)						
Si atoms						
perfect-dc	78.10	79.83	81.61	83.13	83.55	76.55
defective-dc	13.22	12.65	11.89	11.01	10.54	10.46
non-dc	8.68	7.52	6.50	5.86	5.91	12.99
Au atoms						
fcc	39.59	44.27	52.40	53.48	49.05	38.28
hcp	18.17	16.55	12.74	12.41	14.26	13.28
bcc	0.77	0.56	0.37	0.31	0.40	0.42
other	41.47	38.62	34.49	33.80	36.29	48.02

References

(S1) Bal, K. M.; Neyts, E. C. On the time scale associated with Monte Carlo simulations. *The Journal of Chemical Physics* **2014**, *141*, 204104.
